# *Mycobacterium tuberculosis* Exploits Asparagine to Assimilate Nitrogen and Resist Acid Stress during Infection

**DOI:** 10.1371/journal.ppat.1003928

**Published:** 2014-02-20

**Authors:** Alexandre Gouzy, Gérald Larrouy-Maumus, Daria Bottai, Florence Levillain, Alexia Dumas, Joshua B. Wallach, Irène Caire-Brandli, Chantal de Chastellier, Ting-Di Wu, Renaud Poincloux, Roland Brosch, Jean-Luc Guerquin-Kern, Dirk Schnappinger, Luiz Pedro Sório de Carvalho, Yannick Poquet, Olivier Neyrolles

**Affiliations:** 1 Centre National de la Recherche Scientifique, Institut de Pharmacologie et de Biologie Structurale, Toulouse, France; 2 Université de Toulouse, Université Paul Sabatier, Institut de Pharmacologie et de Biologie Structurale, Toulouse, France; 3 Mycobacterial Research Division, MRC National Institute for Medical Research, London, United Kingdom; 4 Dipartimento di Ricerca Traslazionale e delle Nuove Tecnologie in Medicina e Chirurgia, Università di Pisa, Pisa, Italy; 5 Department of Microbiology and Immunology, Weill Cornell Medical College, New York, New York, United States of America; 6 Centre d'Immunologie de Marseille-Luminy (CIML), Inserm UMR 1104, CNRS UMR 7280, Aix-Marseille University UM 2, Marseille, France; 7 Institut Curie, Laboratoire de Microscopie Ionique, Orsay, France; 8 INSERM U759, Orsay, France; 9 Institut Pasteur, Unité de Pathogénomique Mycobactérienne Intégrée, Paris, France; McGill University, Canada

## Abstract

*Mycobacterium tuberculosis* is an intracellular pathogen. Within macrophages, *M. tuberculosis* thrives in a specialized membrane-bound vacuole, the phagosome, whose pH is slightly acidic, and where access to nutrients is limited. Understanding how the bacillus extracts and incorporates nutrients from its host may help develop novel strategies to combat tuberculosis. Here we show that *M. tuberculosis* employs the asparagine transporter AnsP2 and the secreted asparaginase AnsA to assimilate nitrogen and resist acid stress through asparagine hydrolysis and ammonia release. While the role of AnsP2 is partially spared by yet to be identified transporter(s), that of AnsA is crucial in both phagosome acidification arrest and intracellular replication, as an *M. tuberculosis* mutant lacking this asparaginase is ultimately attenuated in macrophages and in mice. Our study provides yet another example of the intimate link between physiology and virulence in the tubercle bacillus, and identifies a novel pathway to be targeted for therapeutic purposes.

## Author Summary

Tuberculosis (TB) is still responsible for nearly 1.3 million deaths annually. There is an urgent need to identify novel drug targets in the tubercle bacillus, *Mycobacterium tuberculosis*, in order to develop novel therapeutics. To proliferate inside its human host, and ensure its spreading, *M. tuberculosis* must adapt its nutritional requirements and metabolism to the molecular environment it encounters during infection. Elucidating the origin, nature, and acquisition mechanisms of the nutrients required by *M. tuberculosis* inside its host may help identify targets for novel antimicrobials. In this study we asked how the TB bacillus acquires nitrogen, a vital constituent of all living organisms, from host tissues. We show the amino acid asparagine to be an important source of nitrogen for the bacillus, and we identify two bacterial proteins, AnsP2 and AnsA, that allow the pathogen to capture and ‘digest’ asparagine, respectively. In addition, we report that asparagine ‘digestion’ allows the pathogen to resist the host immune defense and to survive inside host cells and tissues. This study paves the way for future research into *M. tuberculosis* nitrogen metabolism, and for the development of alternative therapeutic strategies to impair nitrogen acquisition by the bacillus and treat patients with TB.

## Introduction

With nearly 1.3 million lives claimed in 2012, as reported by the World Health Organization, tuberculosis (TB) remains the major cause of death due to a single bacterial pathogen. A better understanding of the interactions between *Mycobacterium tuberculosis*, the etiologic agent of TB, and its human host may help improve current therapies. In particular, unraveling the microbial mechanisms involved in uptake and catabolism of host-derived nutrients required by the pathogen during its life cycle may identify targets for novel antimicrobials [1]–[3].

The TB bacillus is an intracellular microorganism that thrives inside host macrophages. Although *M. tuberculosis* can be found in the host cell cytosol at later stages of infection [4]–[6], the prevailing consensus is that the pathogen resides and multiplies mostly within phagosomes, which fuse poorly with host cell lysosomes and barely acidify (pH∼6.5) [7]–[10]. In macrophages activated by immune cell-derived cytokines, such as interferon (IFN)- γ, and microbial ligands, such as *Escherichia coli*-derived lipopolysaccharide (LPS), the pH of the mycobacterial phagosome drops below 5.5, and mycobacterial growth is constrained to some extent [8], [11], [12]. The ability to block phagosome maturation and avoid lysosomal degradation is considered chief among *M. tuberculosis* virulence strategies, although the molecular mechanisms involved in this process are likely to be multiple and remain yet to be fully elucidated [10]. In addition to being slightly acidic, the mycobacterial phagosome is considered an environment in which nutrient availability is limited [1], [3], [13]. Such multiple stresses typically translate into a marked remodeling of the mycobacterial transcriptional landscape soon after phagocytosis, as supported, for example, by the induction of acid-responsive genes and those involved in utilization of alternative carbon sources, such as host-derived fatty acids and cholesterol [14]–[18]. Carbon metabolism reprogramming, in particular, appears instrumental in mycobacteria adaptation to their host, and a number of studies identified major pathways used by *M. tuberculosis* to gather carbon during infection [19]–[24].

In addition to carbon, nitrogen is an essential component of biomolecules, such as amino acids, nucleotides and organic co-factors. Although several studies provided insight into the regulation mechanisms of the central nitrogen metabolism in *M. tuberculosis*, showing in particular the key role of the glutamine synthetase GlnA1 and its regulator GlnE in this process [25]–[29], the mechanisms by which nitrogen is acquired by the bacillus, and the main nitrogen sources used during infection remain poorly characterized. In this context, we recently reported that *M. tuberculosis* employs the membrane transporter AnsP1/Rv2127 to capture aspartate and exploit this amino acid species as a nitrogen source during infection [30], [31]. Here we further report that the AnsP1 homologue AnsP2 (AroP2/Rv0346c), a predicted asparagine transporter, and that AnsA (Rv1538c), a predicted asparaginase [32], allow asparagine uptake and deamination, respectively. The hydrolysis of asparagine in turn allows *M. tuberculosis* to assimilate nitrogen into downstream metabolites such as glutamate and glutamine. In parallel, this system of asparagine acquisition supports the *in vitro* mycobacterial growth in acidic conditions through ammonia release and pH buffering. Finally, we provide evidence that AnsA is released into the *M. tuberculosis* culture filtrate in vitro and within the mycobacterial phagosome. Thus AnsA is important for phagosome acidification arrest and intracellular survival of the pathogen inside macrophages, ultimately serving as a virulence factor. Collectively, these results provide compelling evidence that asparagine is an important additional source of nitrogen for *M. tuberculosis* during host colonization, and identify AnsA and the asparagine transport system as potential novel targets to be considered for therapeutic purposes.

## Results

### Asparagine catabolism plays a role in *M. tuberculosis* virulence

**Figure 1:**
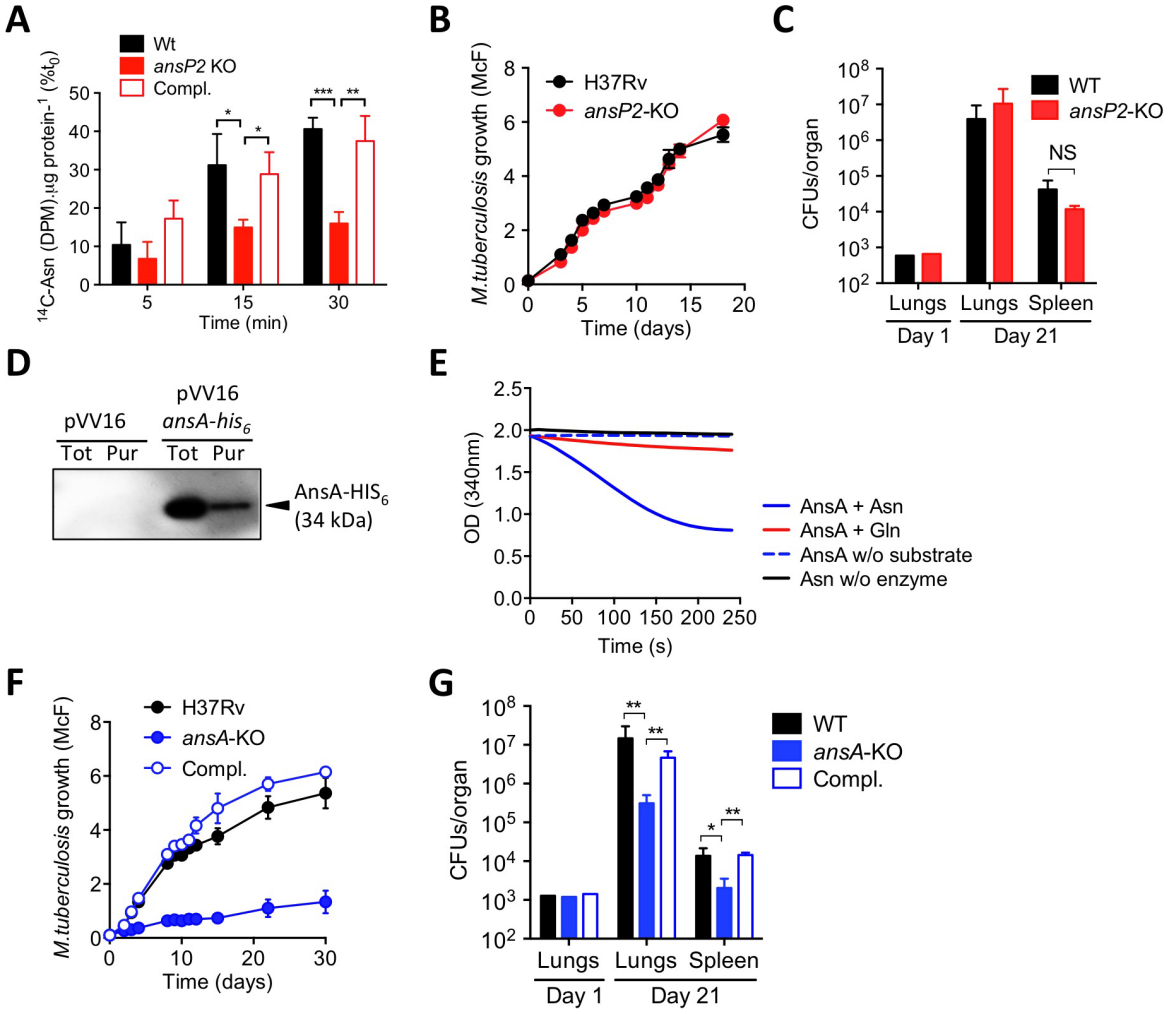
The function and *in vivo* relevance of AnsP2 and AnsA in asparagine utilization in *M. tuberculosis*. (A) U-^14^C-Asn uptake assay with *M. tuberculosis* H37Rv, the *ansP2*-KO mutant and its complemented strains (Compl.). Bacteria previously grown in 7H9 with 5 mM Asn, were harvested and resuspended in an uptake buffer containing a mix of ^14^C-labeled and non-labeled asparagine to obtain a final concentration of 20 µM asparagine. Bacteria were incubated at 37°C and samples were removed and bacteria-associated ^14^C radioactivity was quantified at the indicated time points. Data are expressed as the percentage of the number of disintegrations per minute (DPM) per total protein concentration (^14^C-Asn (DPM). µg protein^−1^), as compared to the values obtained at t_0_. (B) Growth of *M. tuberculosis* H37Rv and the *ansP2*-KO mutant strain in the presence of asparagine as sole nitrogen source. (C) C57BL/6 mice were infected intranasally with 1,000 CFUs *M. tuberculosis* wild type (H37Rv) or the *ansP2*-KO mutant. Three weeks later, lungs and spleen were recovered, homogenized and plated onto agar for CFU scoring. (D) Western blotting analysis of total protein extracts (Tot) or a Ni-NTA purified fraction (Pur) from *M. smegmatis* containing a pVV16 control plasmid (pVV16) or an *ansA-his_6_* cassette cloned into pVV16 (pVV16 *ansA-his_6_*), using an anti-HIS_6_ monoclonal antibody. 1 µg of proteins were loaded in the “Tot” lanes, 0.5 µg of proteins were loaded in the “Pur” lanes. The expected molecular weight of recombinant AnsA-HIS_6_ fusion protein is of 34 kDa. (E) Asparaginase activity, as monitored by NADPH disappearance at OD_340_ (see Materials & Methods), of recombinant AnsA in the presence of asparagine (Asn) or glutamine (Gln). Control reactions lack (w/o) substrate or enzyme. (F) Growth of *M. tuberculosis* H37Rv, the *ansA*-KO mutant strain, and the *ansA*-KO complemented strain (Compl.) in minimal medium containing 5 mM asparagine as sole nitrogen source. (G) C57BL/6 mice were infected intranasally with 1,000 CFUs *M. tuberculosis* wild type (H37Rv), the *ansA*-KO mutant or its complemented strain (Compl.). Three weeks later, lungs and spleen were recovered, homogenized and plated onto agar for CFU scoring. All data are representative of at least two independent experiments. In (A), (C), (F) and (G), data represent mean±s.d. of triplicate samples and were analyzed using the Student's *t* test; *, P<0.05; **, P<0.01; ***, P<0.001. NS, not significant.

Because asparagine is known to be one of the best nitrogen sources used by *M. tuberculosis* in vitro [33], [34], we reasoned that the pathogen may have a transport system in place to scavenge this amino acid from its host. Among putative transporters, AnsP2/Rv0346c became an obvious candidate based on its high primary sequence identity (58%) with the *Salmonella enterica* asparagine transporter AnsP [35]. Moreover, *ansP2* expression is markedly induced in *M. tuberculosis* in the lungs of patients with TB, which may reflect an important role for this putative transporter in a natural setting [36]. In order to evaluate whether AnsP2 transports asparagine, we first performed a ^14^C-asparagine uptake experiment with wild-type *M. tuberculosis* H37Rv and an *ansP2*-deficient mutant strain that we generated by recombineering [30], [37]. In agreement with the functional annotation of AnsP2, we found asparagine transport was partially impaired in the mutant as compared to its wild-type counterpart (Fig. 1A). This phenotype was reversed upon genetic complementation of the mutant strain with an integrative cosmid harboring the *ansP2* gene region (Fig. 1A), thus demonstrating the implication of AnsP2 in asparagine uptake. Based on these results, we hypothesized the *ansP2*-KO mutant should be affected in its ability to grow in the presence of asparagine as sole nitrogen source. Surprisingly, we found the mutant multiplied equally to the wild-type strain under this condition (Fig. 1B), indicating the reduced amount of asparagine imported by the mutant strain (Fig. 1A) was nevertheless sufficient to promote bacterial growth. Moreover, the *ansP2*-KO mutant was not attenuated in immune-competent mice (Fig. 1C). Altogether, while these results identify AnsP2 as an asparagine transporter in *M. tuberculosis*, they also allude to the presence of one or more additional yet to be identified transporter(s) responsible for the uptake of this amino acid species.

Once asparagine is scavenged by the bacillus, we inferred it must undergo an assimilation process carried out by asparaginases, which hydrolyze this amino acid into aspartate and ammonia. Indeed, asparaginase activity was described several decades ago in lysates of various mycobacteria species, including *M. tuberculosis* [38], [39]. In the *M. tuberculosis* genome, a unique gene by the name of *ansA* is predicted to encode an asparaginase [32], and whose homologue was recently proven to hydrolyze asparagine *in vitro* in the closely related attenuated vaccine strain *Mycobacterium bovis* BCG [40]. Building upon these observations, we decided to produce and purify a recombinant HIS_6_-tagged version of AnsA in the *M. tuberculosis*-related fast grower *Mycobacterium smegmatis* in order to evaluate its asparaginase activity. The recombinant enzyme, with a predicted molecular weight of 34 kDa, was immuno-detected both in total bacterial lysate and after purification on a nickel column using an appropriate anti-HIS_6_ antibody (Fig. 1D). The ability of recombinant AnsA to hydrolyze asparagine was then assessed in a coupled enzymatic reaction in which the ammonia generated after asparagine deamination is used in a secondary reaction to form glutamate via a NADPH-dependent glutamate dehydrogenase. Disappearance of NADPH was followed as a marker of asparagine consumption in the reaction mixture, and revealed that AnsA mediates asparagine hydrolysis (Fig. 1E). By contrast, we found AnsA could not hydrolyze glutamine (Fig. 1E), indicating the enzyme is void of any significant glutaminase activity frequently associated to asparaginases [30], [41].

Since AnsA is the only predicted asparaginase in *M. tuberculosis* [32], as opposed to in other bacteria such as *E. coli* [42], we deduced that the genetic inactivation of AnsA should have a significant impact on asparagine metabolism in this species. Given that *ansA* might be essential in *M. tuberculosis* [24], we designed a conditional inactivation strategy to knock this gene out [43] (Figure S1). Unexpectedly, we could readily generate a viable *ansA*-KO mutant strain, revealing *ansA* is not essential in *M. tuberculosis*, as suggested by other studies [44], [45]. The apparent contradiction between the observed viability of the mutant and the essentiality predicted by Griffin *et al.* [24] can be reconciled when considering that for the high density transposon insertion screen asparagine was used as the main nitrogen source in the culture medium; it is most likely that under these conditions an *ansA*-KO mutant is impaired in growth. Consistent with this assumption, and with the observed enzymatic activity of AnsA *in vitro*, we found the growth of the *ansA*-KO mutant was impaired, although not fully abolished, when asparagine was provided as sole nitrogen donor (Fig. 1F). It is likely that the remaining minimal growth of the mutant observed under this condition is due to residual asparagine deamination mediated by other yet to be identified amidases present in *M. tuberculosis*. As a control, the *ansA*-KO mutant replicated equally to the wild-type strain in the presence of another nitrogen source, such as glutamate (Figure S2), suggesting that *ansA* inactivation does not lead to general growth defects. Equally important, we found the *ansA*-KO mutant was impaired in host tissue colonization (Fig. 1G), thus suggesting a role for asparagine catabolism in *M. tuberculosis* virulence.

### AnsP2 and AnsA are involved in nitrogen incorporation from asparagine in *M. tuberculosis*

**Figure 2:**
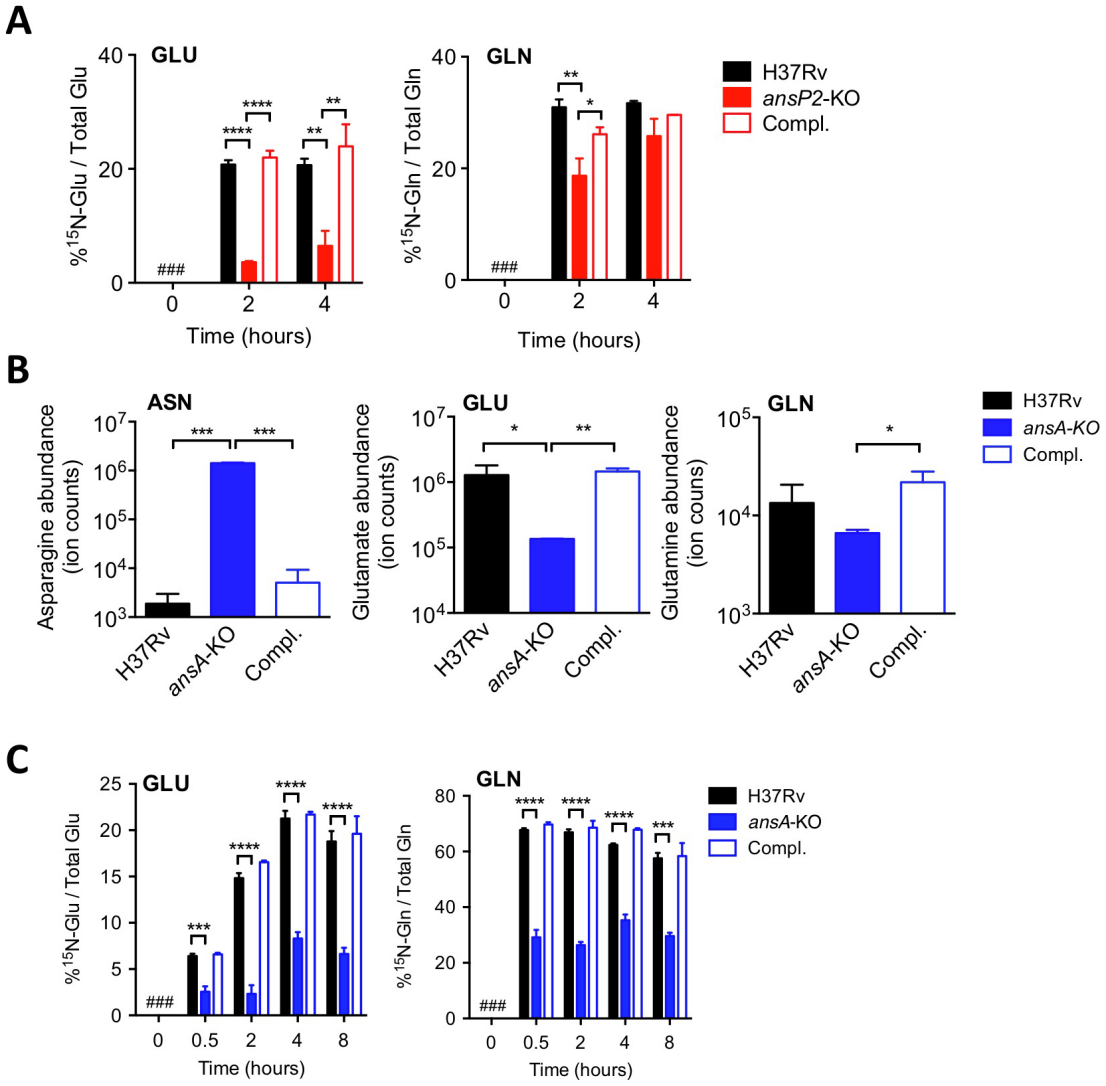
AnsP2 and AnsA are involved in nitrogen incorporation from asparagine in *M. tuberculosis*. (A) Frequency of ^15^N-glutamate (GLU) and ^15^N-glutamine (GLN) detected in the presence of U-^15^N-Asn (2 mM) in *M. tuberculosis* wild type (H37Rv), the *ansP2*-KO mutant and its complemented strain (Compl.). (B) Total asparagine (ASN), glutamate (GLU) and glutamine (GLN) ion counts in *M. tuberculosis* wild type (H37Rv), the *ansA*-KO mutant and its complemented strain (Compl.). (C) Frequency of ^15^N-glutamate (GLU) and ^15^N-glutamine (GLN) detected in *M. tuberculosis* wild type (H37Rv), the *ansA*-KO mutant and its complemented strain (Compl.) cultivated in minimal medium in the presence of 2 mM ^15^N-asparagine as sole nitrogen source. #, not detected. Data represent mean±s.d. of triplicate samples, are representative of two independent experiments, and were analyzed using the Student's *t* test; *, P<0.05; **, P<0.01; ***, P<0.001; ****, P<0.0001.

The results above suggested *M. tuberculosis* exploits asparagine from host tissues during infection to support growth. In agreement with previous studies reporting asparagine is a preferred source of nitrogen in *M. tuberculosis* [33], [34], we found this amino acid does not support mycobacterial growth when provided as sole carbon and energy source (Figure S3). To further understand the role of asparagine assimilation in *M. tuberculosis*, we used targeted metabolomics to follow nitrogen incorporation in the *ansP2*- and *ansA*-KO mutants during growth on ^15^N_2_-labeled asparagine. As compared to wild-type *M. tuberculosis*, we found the *ansP2*-KO mutant was impaired in nitrogen incorporation from asparagine into other amino acids, such as glutamate and glutamine, which serve as initial nitrogen providers in the central nitrogen metabolism; this phenotype was reversed upon genetic complementation with a functional *ansP2* allele (Fig. 2A). Strikingly, total asparagine content of the *ansA*-KO strain at the steady state in a medium containing asparagine as sole nitrogen provider was found ∼1,000-fold higher than in its wild-type and complemented counterparts (Fig. 2B), a likely consequence arising from the impaired asparagine catabolism in the mutant. In line with this hypothesis, the amounts of total (Fig. 2B), as well as newly synthetized (Fig. 2C), glutamate and glutamine were reduced in the mutant strain, further indicating a clear impairment of nitrogen incorporation from asparagine into downstream metabolites in the absence of AnsA. Of notice, nitrogen assimilation from asparagine was not completely abolished in the *ansA*-KO mutant, and this phenotype paralleled the residual growth of the mutant in the presence of asparagine reported above (Fig. 1F). Altogether, these results reveal that asparagine-derived nitrogen is fully assimilated in *M. tuberculosis*, and that AnsP2 and AnsA are involved in this process.

### Asparagine catabolism in *M. tuberculosis* mediates resistance to acid stress and intracellular survival

**Figure 3:**
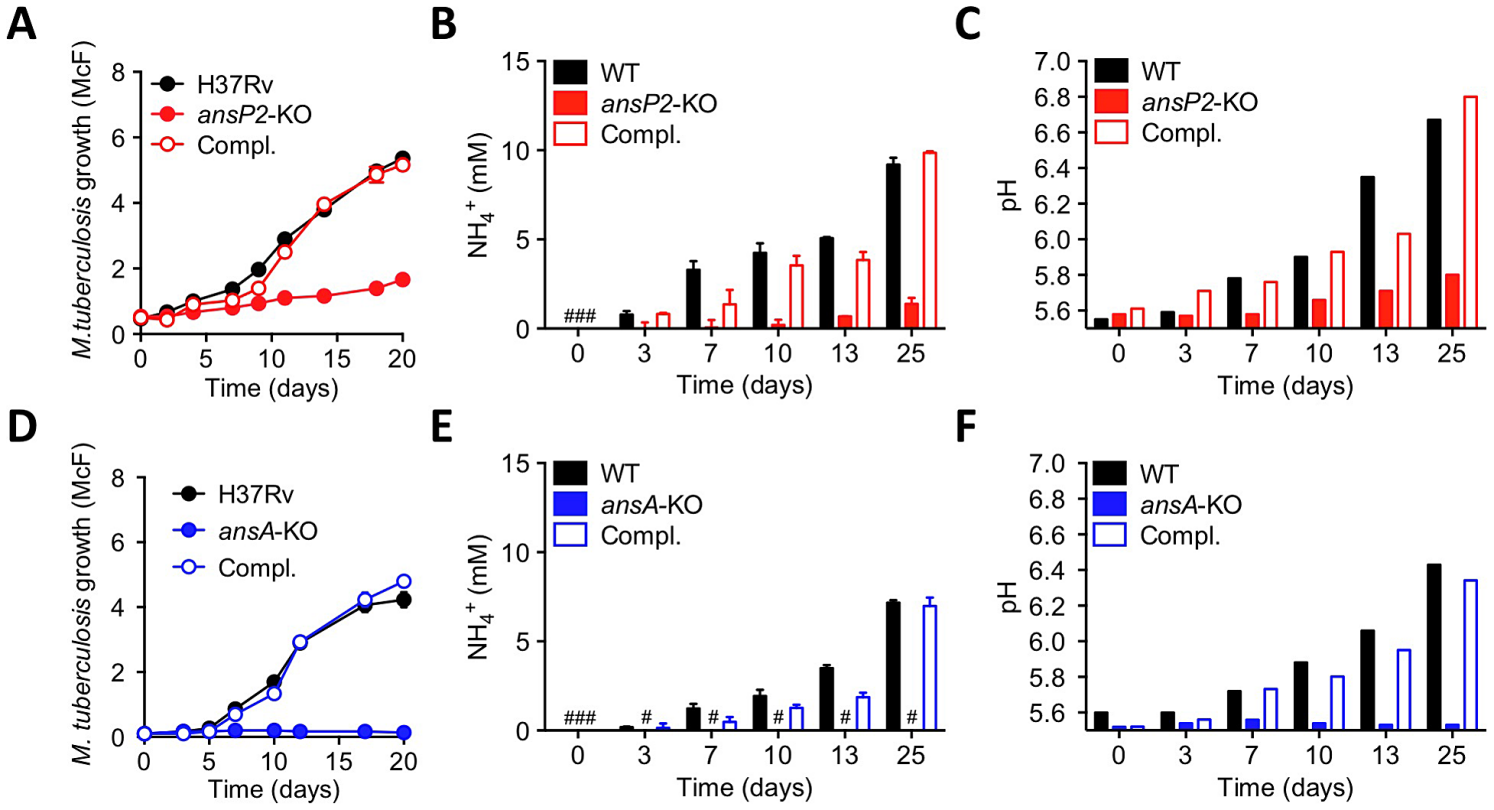
Varied requirement of AnsP2 and AnsA for *M. tuberculosis* resistance to acid stress in vitro. (A–C) Growth (A), culture supernatant NH_4_^+^ concentration (B) and pH (C) of *M. tuberculosis* H37Rv, the *ansP2*-KO mutant strain, or the *ansP2*-KO complemented strain (Compl.) at acidic pH (5.5) in the presence of asparagine as sole nitrogen source. (D–F) Growth (D), culture supernatant NH_4_^+^ concentration (E) and pH (F) of *M. tuberculosis* H37Rv, the *ansA*-KO mutant strain, or the *ansA*-KO complemented strain (Compl.) at acidic pH (5.5) in the presence of asparagine as sole nitrogen source. Data represent mean±s.d. of triplicate samples and are representative of at least three independent experiments. #, not detected.

A recent study reported asparagine is the best among the few amino acids that can support *M. tuberculosis* resistance to acid stress [46]. This feature most likely relies on the specific release of the weak base ammonia and subsequent pH buffering that accompany asparagine consumption [46]. Building upon this observation, we found the growth of the *ansP2*-KO strain, in the presence of asparagine as sole nitrogen provider, was greatly reduced at pH 5.5 as compared to the wild-type and complemented strains (Fig. 3A). This phenotype correlated with a markedly diminished capacity of the mutant to secrete ammonia and neutralize pH of the culture medium (Fig. 3B,C). In the same conditions, the phenotypes of the *ansA*-KO mutant were even more pronounced. Indeed, mycobacterial growth, asparagine-mediated ammonia secretion and pH buffering were totally abolished in the absence of AnsA (Fig. 3D–F). In line with these results, nitrogen assimilation from asparagine into glutamate and glutamine was fully abrogated in the *ansA*-KO mutant at acidic pH (Figure S4A). In order to rule out the possibility that the observed defect in ammonia secretion and pH buffering in the *ansA*-KO mutant was due to the growth defect of the mutant at acidic pH, we repeated the experiment reported in Fig. 3D–F using a more dense bacterial suspension and a shorter time course, with and without asparagine as sole nitrogen source. We resuspended bacteria at an OD_600_ of 1.5 in acidic culture medium and measured ammonia secretion and pH at 0, 2, 4, 18 and 24 hours after inoculation. In these conditions, the *ansA*-KO mutant was still completely impaired in ammonia secretion and pH buffering, as compared to its wild-type and complemented counterparts (Figure S4B–D). Collectively, these results unequivocally demonstrate that, in particular in an acidic environment, asparagine catabolism partially requires AnsP2 and is strictly dependent on AnsA to sustain *M. tuberculosis* growth in the presence of asparagine. These results also underline that asparagine hydrolysis, ammonia release, pH buffering and growth in acidic conditions are intrinsically linked molecular events in *M. tuberculosis*.

**Figure 4:**
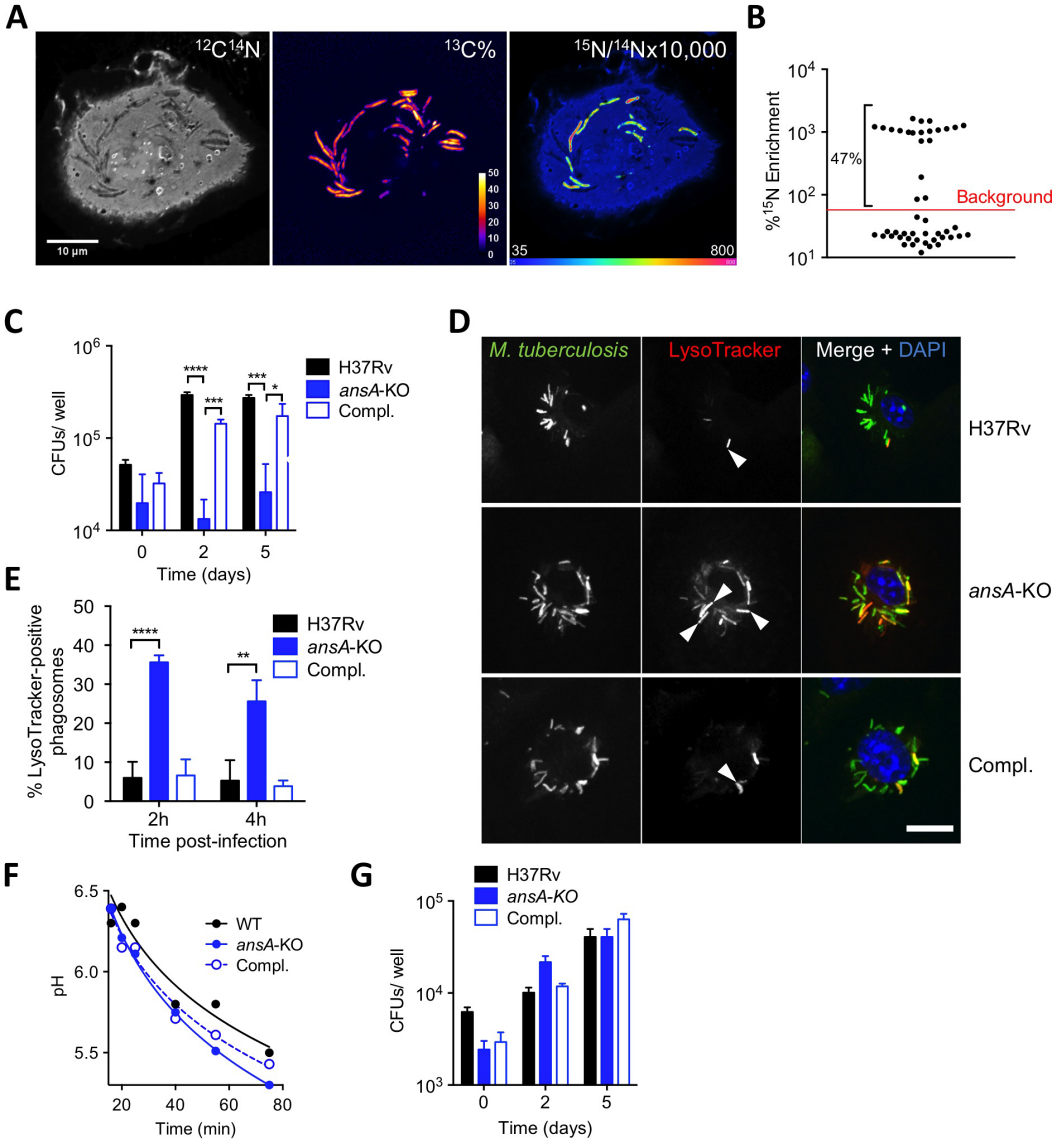
Requirement of AnsP2 and AnsA for *M. tuberculosis* resistance to acid in host macrophages. (A,B) Exogenous asparagine accumulates in the mycobacterial phagosome. (A) Images from a representative infected cell showing the locations of *M. tuberculosis* (^13^C% map, middle) and ^15^N-asparagine uptake (^15^N/^14^N ratio map, right), as derived from secondary ion mass spectrometry (SIMS) analysis of *M. tuberculosis* H37Rv-infected mouse bone marrow-derived macrophages (BMMs). ^13^C-labeled bacteria were used to infect BMMs at a multiplicity of infection of 10 bacteria per cell. At 20 h post-infection, infected cells were pulsed for 4 h with 5 mM ^15^N_1 (amine)_-asparagine, and ^13^C and ^15^N isotope proportions were analyzed. (B) Quantification of ^15^N isotope enrichment in surface areas chosen in the intracellular ^13^C-labeled bacteria; “background” indicates the level of enrichment measured in the host cell cytoplasm. For more details about the technique, see [30]. (C) IFNγ- and LPS-activated BMMs were infected with *M. tuberculosis* wild type (H37Rv), the *ansA*-KO mutant or its complemented strain (Compl.) at a multiplicity of infection (MOI) of 0.1 bacterium/cell for 4 h at 37°C. Cells were washed and further incubated with fresh medium for 0, 2 or 5 days. At the indicated time-points, cells lysates were plated for CFU scoring. (D) Confocal microscopy analysis of activated BMMs infected for 1 h with Alexa Fluor 488-labeled *M. tuberculosis* wild type (H37Rv), the *ansA*-KO mutant or its complemented strain (Compl.) (green), and stained with LysoTracker Red DND-99 (red) and DAPI (blue) to visualize nuclei. Bar represents 10 µm. Arrowheads point to example phagosomes considered positive for LysoTracker staining. (E) Quantification of LysoTracker-positive phagosomes in samples prepared as in (h) 2 or 4 h after infection. Colocalisation events were recorded in ≈300 phagosomes observed in ≈10 different fields. (F) Phagosomal pH measured by flow cytometry in activated BMMs infected with *M. tuberculosis* wild type (H37Rv), the *ansA*-KO mutant or its complemented counterpart (Compl.). (G) Cells were pre-incubated with 100 nM bafilomycin A1 for 1 h, infected as in (C) and bafilomycin A1 was removed after 24 h. All data are representative of at least three independent experiments. In (C), (E) and (G), data represent mean±s.d. of triplicate samples, and were analyzed using the Student's *t* test. *, P<0.05; **, P<0.01; ***, P<0.001; ****, P<0.0001.

We next evaluated to what extent the sensitivity of our mutants to acid stress may impact their ability to survive in an acidic phagosome and to parasitize host macrophages. We first assessed whether asparagine can access the mycobacterial phagosome inside infected cells. To this aim, we employed secondary ion mass spectrometry (SIMS), a method that allows the visualization of isotopic labeling and metabolites in biological samples with sub-micrometer resolution. We infected mouse bone marrow-derived macrophages (BMMs) with ^13^C-labelled *M. tuberculosis* H37Rv for 20 hours, and pulsed the infected cells with ^15^N-asparagine for 4 h before SIMS analysis. Our data clearly indicate that exogenously provided asparagine accumulates in the mycobacterial phagosome (in ≈50% of them in Fig. 4A), as compared to the host cell cytosol (Fig. 4A,B). Regarding mycobacterial growth, the *ansP2*-KO mutant was found not affected in its ability to survive in IFNγ- and LPS-activated BMMs, in which the pH of the mycobacterial phagosome readily drops below 5.5 ([8]; Figure S5). This result correlates with the remaining amount of ammonia secretion observed in this mutant (Fig. 3B). On the other hand, the intracellular survival of the *ansA*-KO strain was strongly impaired in activated BMMs (Fig. 4C). Strikingly, labeling of infected cells with the acidotropic dye LysoTracker and phagosomal pH measurement at early time-points after infection revealed that the phagosomes harboring the *ansA*-KO mutant acidified more readily compared to those containing the wild type or complemented strains (Fig. 4D–F). In line with this finding, we also found that V-ATPase, the proton pump responsible for phagosomal acidification, accumulated in larger amounts in phagosomes containing the *ansA*-KO mutant than in vacuoles containing its wild-type or complemented counterparts (Figure S6A,B). Consistent with these observations, treatment of BMMs with bafilomycin A1, a specific V-ATPase inhibitor preventing phagosome acidification [47], restored the ability of the *ansA*-KO mutant to multiply intracellularly (Fig. 4G). Whether the attenuation phenotype of the *ansA*-KO mutant inside macrophages is a cause or a consequence of impaired asparagine hydrolysis and subsequent reduced ammonia production and pH buffering capacity is difficult to delineate, as these molecular events are intrinsically linked one to each other; nevertheless, our results clearly establish AnsA is required for intracellular survival of *M. tuberculosis*.

### AnsA is a secreted asparaginase in *M. tuberculosis*

**Figure 5:**
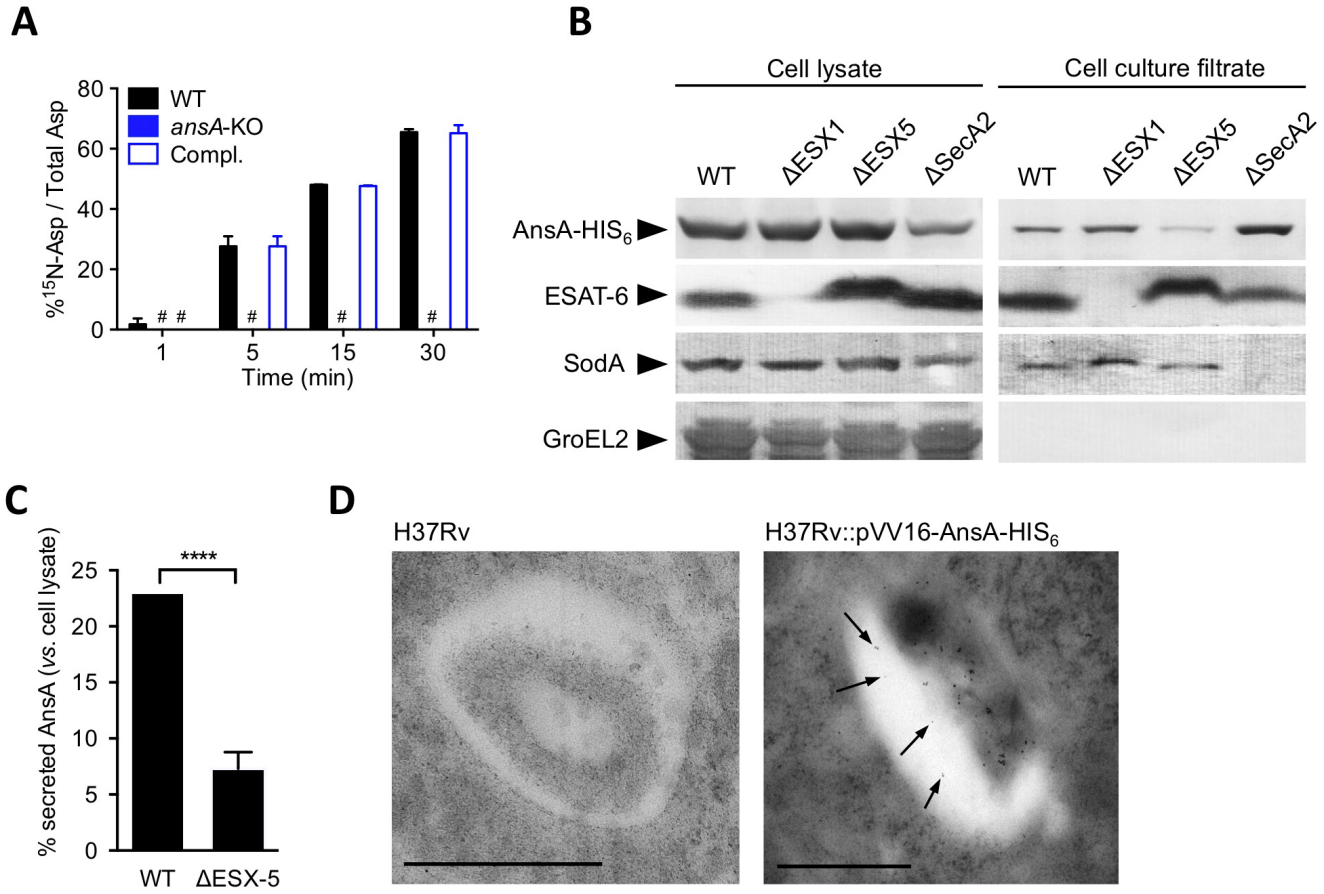
In vitro expression and localization of recombinant AnsA-His_6_ in *M. tuberculosis* wild-type and ESX-1, ESX-5 or SecA2 mutant strains. (A) Asparaginase activity in the supernatant of *M. tuberculosis ansA*-KO mutant and its wild-type and complemented counterparts. Fifty mL of cultures (OD_600_≈0.5) were concentrated 50 times. The concentrates were incubated with ^15^N_1 (amine)_-asparagine at 37°C; at the indicated time points, the reaction mixtures were mixed with an equal volume of acetonitrile:methanol:water (2∶2∶1) and analyzed by MS for the presence of ^15^N-aspartate. (B) Expression and secretion of AnsA-His_6_ in *M. tuberculosis* wild-type and ΔESX-1 [63], ΔESX-5 [62], ΔSecA2 (Bottai *et al.* unpublished data) mutants. Fifteen µg of total cell lysates or culture filtrate proteins from the different mycobacterial strains were subjected to SDS-PAGE and tested in Western blotting by using a mouse anti-His_6_ monoclonal antibody. As control, samples were tested with the anti-ESAT-6 and anti-SodA monoclonal antibodies. Preparations were also tested with the anti-GroEL2 antibody, which was used for lysis control. As expected, cell lysates and cell culture filtrates from all *M. tuberculosis* strains transformed with the empty vector p-VV16 were negative when tested with the anti-His_6_ monoclonal antibody (data not shown). (C) Quantification of the relative expression of AnsA-HIS_6_ in the culture filtrate, as compared to in the cell pellet, in the WT and ESX-5-KO strains. Data represent mean±s.d. of triplicate samples, and were analyzed using the Student's *t* test. ****, P<0.0001. (D) Ni-NTA-Nanogold detection of AnsA-HIS_6_ by electron microscopy in ultrathin sections of *M. tuberculosis*-infected BMMs. Bar indicates 0.5 µm; arrowheads indicate gold particles in the phagosomal lumen.

Strikingly, *M. tuberculosis* AnsA is more similar to the periplasmic (type II) asparaginase AnsB than to the cytosolic (type I) enzyme AnsA from *E. coli* (35% *vs*. 28% identity, respectively) [42], suggesting AnsA might be a secreted asparaginase in *M. tuberculosis*. We addressed this important issue using different and complementary approaches: i) quantification of asparaginase activity in cell-free culture supernatants by mass spectrometry (MS); ii) immune-detection of AnsA-HIS_6_ fusion protein in culture filtrates from recombinant *M. tuberculosis* strains; iii) analysis of AnsA secretion in phagosomes of *M. tuberculosis*-infected macrophages by electron microscopy (EM). We incubated bacterial culture supernatants from the wild type, *ansA*-KO and complemented strains with ^15^N-asparagine and monitored ^15^N-aspartate production by MS. Our data revealed that an asparaginase activity could be detected in the *M. tuberculosis* culture supernatant, unless *ansA* was genetically inactivated (Fig. 5A). Consistently, we immuno-detected the AnsA-HIS_6_ fusion protein in culture filtrate, as well as in the cell pellet, of recombinant *M. tuberculosis* by Western blotting (Fig. 5B). As expected, the strictly cytosolic protein GroEL2 was detected in the cell pellet only, indicating the absence of bacterial lysis. Because AnsA does not contain a classical signal peptidase I cleavage site in its N-terminal end, we investigated whether alternative secretion systems, such as the SecA2 secretion system [48], [49] or the ESX-1 and ESX-5 type VII secretion systems [50] might be involved in AnsA secretion. To this aim, we transformed ESX-1, ESX-5 and SecA2-KO mutants with the AnsA-HIS_6_ fusion-encoding plasmid, purified the culture filtrate from exponentially growing cultures, and immuno-detected the fusion protein using the anti-HIS_6_ antibody. Our data indicate that AnsA secretion is independent of SecA2 and ESX-1 (Fig. 5B). As a control, the SecA2-dependent protein SodA was not detected in the supernatant of the SecA2-KO strain. Surprisingly, secretion of AnsA was impaired in the ESX-5 mutant (Fig. 5B,C), indicating the involvement of this type VII secretion system in the secretion of the enzyme. In order to evaluate whether AnsA is also secreted in the phagosomal lumen inside macrophages, we used EM and Ni-NTA-Nanogold to detect AnsA-HIS_6_ in ultrathin sections of cells infected with *M. tuberculosis* carrying or not the AnsA-HIS_6_ fusion-encoding genetic construct. Gold particles were detected in the phagosomal lumen, strongly suggesting AnsA is also secreted in host cells during infection (Fig. 5D).

Collectively, this study puts forward an acquisition system for asparagine that not only protects against phagosomal acidification, but also serves to assimilate nitrogen from this amino acid species, with a central role for the asparaginase AnsA in enhancing the fitness of *M. tuberculosis* during host colonization.

## Discussion

**Figure 6:**
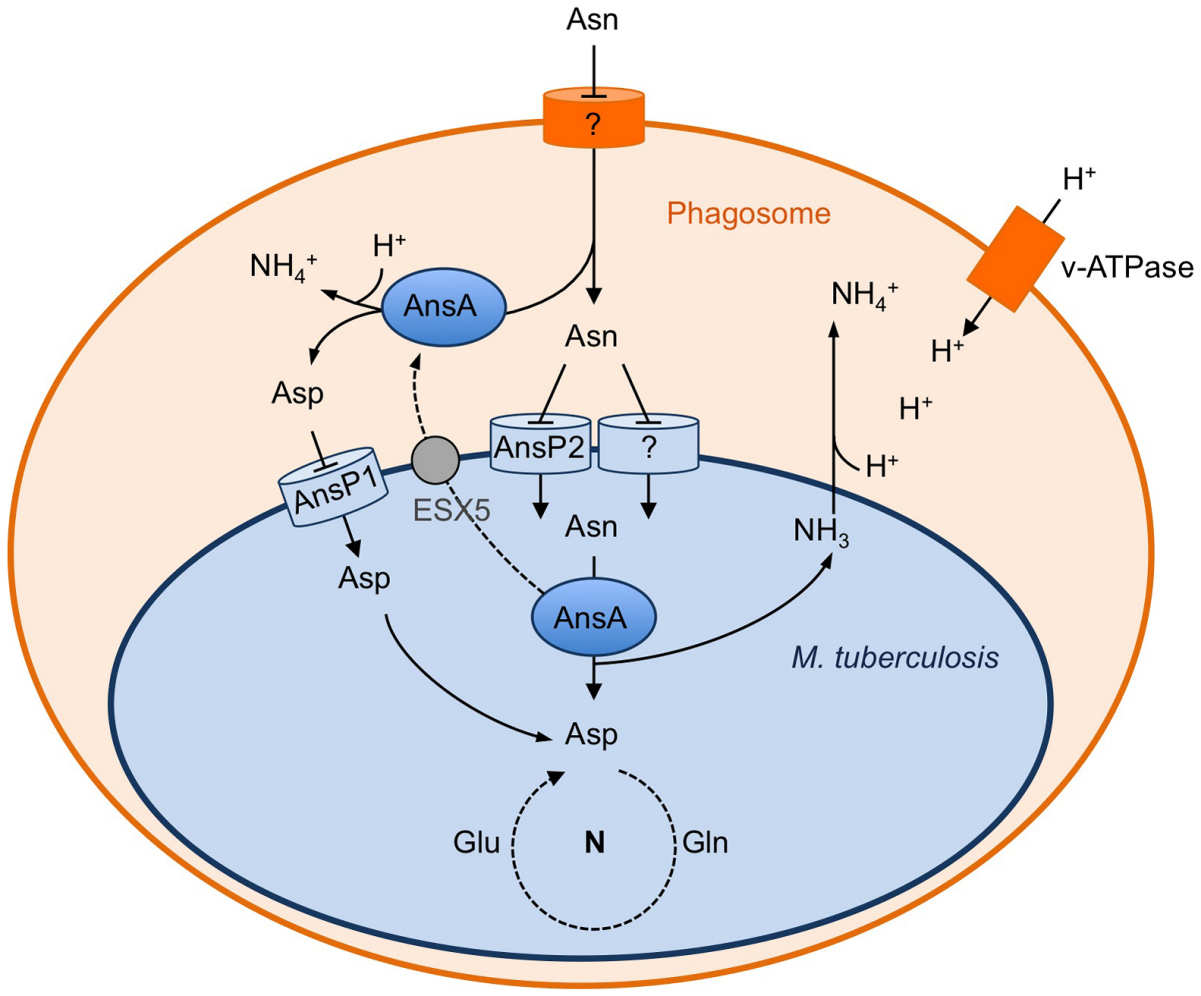
Schematic representation of the role of asparagine catabolism in nitrogen incorporation, resistance to acid and intracellular survival. Within macrophages, asparagine enters the *M. tuberculosis* phagosome through an unknown mechanism. Asparagine is captured by *M. tuberculosis* through AnsP2 and one or more other yet to be identified transporter(s), and hydrolyzed by cytosolic AnsA resulting in nitrogen assimilation into glutamine and glutamate, and release of ammonia. AnsA is secreted in the lumen of the phagosome through, at least in part, an ESX-5-dependent mechanism. AnsA can also hydrolyze asparagine in the lumen of the phagosome, resulting in the production of aspartate and ammonia. Aspartate is imported by AnsP1 [30] for nitrogen assimilation. In the phagosomal lumen, ammonia reacts with protons transported by the V-ATPase to form ammonium ions allowing phagosomal pH buffering.

Identifying the nutrients used by *M. tuberculosis* to assimilate essential elements, such as carbon and nitrogen, is key to understanding host-pathogen interactions in TB. In this context, we recently reported that aspartate is a key nitrogen source used by *M. tuberculosis* during infection [30], [31]. Here we further show that asparagine can serve as an additional source of nitrogen for the pathogen through transport by the amino acid permease AnsP2, and subsequent hydrolysis by the asparaginase AnsA (Fig. 6). Furthermore, our results establish a unique link between mycobacterial physiology and virulence since we show AnsA has a dual function in both nitrogen assimilation and in protection against acid stress in vitro and inside host cells (Fig. 4). Our results are most likely relevant from a physiological viewpoint since asparagine is present at 50–60 µM in the human plasma [51], and is 2- to 4-fold more concentrated in white blood cells [29], [51], [52]. In addition, we further show here that asparagine accumulates in the mycobacterial vacuole inside infected macrophages. Altogether, these observations indicate that asparagine is most likely readily accessible to *M. tuberculosis* during infection in vivo.

Regarding asparagine uptake in *M. tuberculosis*, it is clear from the present study that one or more transporter(s) complement the function of AnsP2, since the *ansP2*-KO mutant was only partially impaired in nitrogen incorporation from asparagine in vitro, and it was not attenuated inside host cells and *in vivo*. The AnsP2 paralogue AnsP1 (72% identity) is an obvious candidate to fulfill this function [32]. However, we previously reported that the *M. tuberculosis ansP1*-KO mutant grows and incorporates asparagine equally well, compared to the wild-type strain, when grown on asparagine as sole nitrogen source [30]. Furthermore, we found the *ansP1*-KO mutant transports asparagine to the same extent as the wild-type strain in vitro (data not shown). In addition to AnsP1, two other putative amino acid transporters, namely CycA/Rv1704c and GabP/Rv0522 [32], show some similarity with AnsP2 (38% and 34% identity, respectively) and may contribute to asparagine transport. The construction of multiple mutants inactivated in two or more of these candidates will be required in order to uncover the complete asparagine transport machinery in *M. tuberculosis*, which will be the purpose of future study. Nevertheless, our results identify AnsP2 as an important asparagine transporter in *M. tuberculosis*, in particular at acidic pH.

Beyond the complexity of asparagine uptake, further efforts should be allocated to deciphering the exact contribution of AnsA to mycobacterial virulence. Whether attenuation of the *ansA*-KO mutant in vivo is due to its inability to counteract phagosome acidification and/or to incorporate nitrogen from asparagine resulting in an impaired fitness will require careful investigation; however such an investigation will be made difficult by the intrinsically linked nature of the asparagine hydrolysis, ammonia release and pH buffering phenomenons in *M. tuberculosis*, as revealed by our study and in a previous report [46]. In this context, it is worth noticing that, like AnsA, another mycobacterial hydrolase, namely the urease, was proposed to play a part both in nitrogen acquisition and in counteracting phagosome acidification through hydrolysis of urea and subsequent release of ammonia [53]–[55]. However, unlike for AnsA, mycobacterial mutants deficient in urease production are barely impaired in intracellular survival and their capacity to persist or multiply in vivo is not affected [53]–[55].

Finally, a role for asparaginase in virulence of other bacterial pathogens, including *Helicobacter pylori, Campylobacter jejuni* and *Salmonella typhimurium*, has been reported [56]–[60]. In these species, asparaginase is secreted into the periplasm and is thought to contribute to host colonization either through direct microbial asparagine utilization in vivo [56], or through indirect starvation-mediated exhaustion of immune cells following asparagine depletion in infected tissues [57]–[60]. The *M. tuberculosis* asparaginase AnsA does not contain any detectable signal sequence in its N-terminal end. Yet, we show that this enzyme is secreted in vitro and inside infected macrophages through an alternative SecA2- and ESX-1-independent pathway that relies, at least partially, on the ESX-5 type VII secretion system [50]. The exact mechanism of AnsA secretion, and the extent to which ESX-5 is involved in this process, remain to be further delineated; however it is worth noticing that AnsA contains two sequences resembling the ESX secretion signal consensus YXXXD/E: Y^207^PGSD^211^ and Y^282^GPGHD^287^ [61]. Whether these motifs play a part in AnsA secretion will need to be understood; equally important will be to understand the role of AnsA beyond nitrogen supply to the pathogen, possibly in asparagine depletion and immune cell exhaustion, as reported for other pathogens [57].

In conclusion, our study provides yet another example of the tight connections forged throughout evolution between physiology and virulence in microbial pathogens. It also highlights the need to further explore the expanding field of metabolism and infection in order to accelerate the identification and validation of novel strategies to combat infections and disease.

## Materials & Methods

### Mycobacteria and culture conditions

Mycobacteria were grown at 37°C in Middlebrook 7H9 medium (Difco) supplemented with 10% albumin-dextrose-catalase (ADC, Difco) and 0.05% Tween-80 (Sigma), or on Middlebrook 7H11 agar medium (Difco) supplemented with 10% oleic acid-albumindextrose- catalase supplement (OADC, Difco). When required, kanamycin, hygromycin, streptomycin (50 µg/mL) or zeocin (25 µg/mL) were added to the culture media. The ESX-1 and ESX-5 mutants have been described previously [62], [63]. A SecA2 mutant carrying a kanamycin-inactivated copy of the *secA2*/rv1821 gene was constructed using a similar strategy based on the ts-SacB technology (Bottai *et al.* Unpublished data). For growth tests with asparagine as a carbon source, bacteria were grown in Sauton's modified medium (pH 6.5–7.0) containing, 0.5 g/L KH_2_PO_4_, 0.5 g/L MgSO_4_, 50 mM asparagine, 0.05% tyloxapol (Sigma) and supplemented with or without 10 g/L glycerol and 15 mM (NH_4_)_2_SO_4_. For growth tests with asparagine as sole nitrogen source, bacteria were grown in Sauton's modified medium containing 0.05% Tween-80, 0.5 g/L KH_2_PO_4_, 0.5 g/L MgSO_4_, 2 g/L citric acid, 10 g/L glycerol and 5 mM asparagine prepared in tap water and neutralized to pH 7.0 or pH 5.5 with NaOH before autoclaving. Cultures were performed in triplicate in glass tubes and bacterial growth was monitored measuring turbidity (in McFarland units) over time using a Densimat apparatus (BioMerieux).

### Construction of *ansP2*-KO and *ansA*-KO mutants and complemented strains

The *ansP2*-KO mutant strain of *Mycobacterium tuberculosis* H37Rv containing a disrupted *ansP2* (Rv0346c)::KanR allele was constructed by allelic exchange using recombineering [30], [37]. H37Rv:pJV53 was grown in 7H9-ADC-Tween 80 in the presence of hygromycin until mid-log phase and expression of the recombineering enzyme was induced by 0.2% acetamide (Sigma) overnight at 37°C. After induction, electrocompetent bacteria were prepared. Electroporation was performed with a linearized fragment of a kanamycin resistance cassette-interrupted *ansP2* gene flanked with homologous regions (400–500 bp length). After 72 h incubation at 37°C, bacteria were plated onto 7H11-OADC agar medium in the presence of kanamycin. For the complementation of the *ansP2*-KO strain, we used the pYUB412-derived integrative cosmid I541, which contains a hygromycin resistance cassette and harbors a fragment encompassing the region 398 to 432 kbp in the genome of *M. tuberculosis* H37Rv. For the *ansA*-KO strain construction, a second copy of the *ansA* gene was first integrated in the chromosome of wild type H37Rv at the bacteriophage insertion site *attL5*. For this, we used the plasmid pGMCS-Puv15-*ansA* which contains the *ansA* gene under the control of the Psmyc promoter and a streptomycin resistance cassette[64]. After selection of streptomycin resistant clones, the original *ansA* gene was disrupted using a linearized digestion fragment of kanamycin resistance cassette-interrupted *ansA* gene flanked with homologous regions (450–600 bp length). The additional copy of *ansA* was then deleted by replacing pGMCS-Puv15-*ansA* with the plasmid pGMCZq17, which contains a zeocine resistance cassette. Selection of a zeocin resistant clone resulted in an *ansA*-KO strain and proved that *ansA* is not essential. For complementation of the *ansA*-KO strain, the pYUB412-derived integrative cosmid I16 encompassing the region 1,719 to 1,756 kbp in the genome of *M. tuberculosis* H37Rv, and containing a hygromycin resistance cassette was used.

### ^14^C-Asparagine uptake experiment

Asparagine uptake experiments were carried out as described elsewhere with minor modifications [46]. Briefly, bacteria were grown in Middlebrook 7H9 containing 0.05% Tween 80 and asparagine (5 mM) at 37°C. Bacteria were harvested by centrifugation when an OD600∼0.5 was reached. Bacterial pellets were washed twice in uptake buffer [50 mM Tris-HCl pH 6.9, 15 mM KCl, 10 mM (NH_4_)_2_SO_4_, 0.05% Tween 80] and resuspended in the same buffer. Radiolabeled ^14^C-asparagine (PerkinElmer) and non-labeled asparagine (Sigma) were mixed (3∶1) and added to 5 mL of cell suspensions to obtain a final concentration of 20 µM asparagine. The mixtures were incubated at 37°C and 250 µL of samples were removed at the indicated time points. Bacteria were collected on a 0.45 µm Spin-X centrifuge tube filter (Costar) by mixing with an equal volume of 10% paraformaldehyde (Polyscience, Inc) containing 0.1 M LiCl (Sigma). Filters radioactivity was determined in a liquid scintillation counter (Packard). The uptake rate was expressed in desintegration per minute (DPM) per total protein concentration (^14^C-Asn (DPM). µg protein^-1^).

### Ammonium and pH measurement

Bacteria were grown in supplemented 7H9 with 0.05% Tween-80 until OD_600_∼1. 10 mL of cultures were removed and washed twice with DPBS and used for inoculation of 200 mL Sauton's modified medium containing 0.05% Tween-80, 0.5 g/L KH_2_PO_4_, 0.5 g/L MgSO_4_, 2 g/L citric acid, 10 g/L glycerol and 5 mM asparagine prepared in tap water and buffered to pH 5.5 with NaOH before autoclaving. Bacteria were incubated at 37°C and, at indicated time points, 1 mL of culture was removed and centrifuged at 1,300 rpm for 2 min to collect supernatants. To determine ammonium concentration, supernatants were diluted 4-fold in DPBS and 50 µL of diluted samples were mixed in a 96 plate with 50 µL of Nessler's reagent (Fluka) and incubated for 20 min at room temperature. 100 µL of NaOH were then added to stop the reaction prior to measurement of OD_520_ using a µQuant apparatus (BIO-TEK instruments, Inc). pH was measured directly in 1 mL of culture supernatant using a pH-meter.

### Expression, purification and measure of enzymatic activity of recombinant HIS_6_-tagged AnsA

The *ansA* gene was cloned into a pVV16 vector allowing the constitutive expression of C-terminus HIS_6_-tagged fusion proteins under the control of a GroEL2 promoter and carrying kanamycin and hygromycin resistance cassettes. The pVV16 *ansA-his_6_* vector was electroporated into the non-pathogenic fast-grower *Mycobacterium smegmatis* mc^2^155 strain and clones containing pVV16 *ansA-his_6_* were selected on solid medium containing kanamycin and hygromycin.

At OD_600_∼1,5, 15 mL of cultures were centrifuged and washed with DPBS and bacteria were resuspended in 1 mL of lysis buffer containing 50 mM NaH_2_PO_4_ pH 8.0, 300 mM NaCl and 10 mM imidazole and broken with glass beads (0.1–0.25 mm) for 10 min at 30 m/s using a Bead Beater apparatus (Retscher, BioBrock scientific). AnsA-HIS_6_ protein was purified from 600 µL of lysates using the Ni-NTA Spin kit (QIAGEN) and eluted in an elution buffer containing 50 mM NaH_2_PO_4_, 300 mM NaCl and 400 mM imidazole at pH 8. AnsA-HIS_6_ purified fraction was quantified using the Bradford method.

For enzymatic tests, we used the L-Asparagine/L-Glutamine/Ammonia Assay Kit (Megazyme) following manufacturer's recommendations and using asparagine or glutamine (final concentration 0.6 mM) as substrates. The buffer used in this assay contains glutamate dehydrogenase, NADPH and 2-oxoglutarate, so that enzymatic activities were measured by following the disappearance of NADPH along time as an indirect indication of asparagine deamination at 340 nm using a SAFAS Monaco mc^2^ spectrophotometer and the SAFAS SP 2000 software.

### Preparation of culture filtrates and total lysates from *M. tuberculosis* strains and immunoblotting

The procedures were as previously described [62]. Immunoblot analyses were carried out with mouse monoclonal antibodies raised against EsxA (Hyb76-08, Antibodyshop, BioPorto Diagnostics) or SodA (NR-13810, clone CS-18, produced in vitro, received from BEI resources N°SOE76725), or with a rabbit polyclonal antibody against the HIS_6_ tag (eBioscience). As control, culture supernatants were also analyzed by Western blot for the presence of GroEL2 (anti-GroEL2 monoclonal antibody, Colorado State University, NIH, NIAID contract N°AI75320).

### Metabolite extraction experiments

Bacteria were cultivated to an OD_600_ of 1 in 7H9-0.05% Tween-80. Bacteria were centrifuged and resuspended in DPBS (3-fold concentration). 1 mL was transferred to a filter (Fisher) mounted on a filtration device (Fisher) and connected to a trap and vacuum line. Filters were transferred to a 7H10 based agar medium (Sigma) supplemented with asparagine (2 mM) or to solid media containing 0.5 g/L KH_2_PO_4_, 0.5 g/L MgSO_4_, 2 g/L citric acid, 10 g/L glycerol, aspartate (2 mM) and 1.5% agar (Invitrogen) prepared in tap water and neutralized to pH 6.5–7.0 with NaOH before autoclaving. Plates were incubated for 5 days at 37°C. Three filters were used per strain and time point. For labeling experiments, filters were transferred on equivalent plates where aspartate was replaced by ^15^N_2_-asparagine (2 mM, Sigma, Purity 98 atom % ^15^N) and incubated for 0.5, 2, 4 or 8 h at 37°C. At each time point, filters were plunged into 1 mL acetonitrile/methanol/water (2∶2∶1, v/v/v) mixture at −40°C. Bacteria were then broken by glass beads using a bead-beater (5 min at 30 m/s). After centrifugation, supernatants were collected and filtered through a Spin-X column 0.2 µm at 14,000 rpm for 15 min. Extracts were stored at −80°C before analysis.

### Liquid chromatography-mass spectrometry

Aqueous normal phase liquid chromatography was performed using an Agilent 1200 LC system equipped with a solvent degasser, binary pump, temperature-controlled auto-sampler (set at 4°C) and temperature-controlled column compartment (set at 20°C), containing a Cogent Diamond Hydride Type C silica column (150 mm×2.1 mm; dead volume 315 µl), from Microsolv Technology Corporation. Flow-rate of 0.4 ml/min was used. Elution of polar metabolites was carried out using gradient 3[65]. Briefly, solvent A consists in deionized water (Resistivity ∼ 18 MΩ cm), 0.2% acetic acid and solvent B consists in acetonitrile and 0.2% acetic acid, and the gradient as follows: 0 min 85% B; 0–2 min 85% B; 2–3 min to 80% B; 3–5 min 80% B; 5–6 min to 75% B; 6–7 min 75% B; 7–8 min to 70% B; 8–9 min 70% B; 9–10 min to 50% B; 10–11 min 50% B; 11–11.1 min to 20% B; 11.1–14 min hold 20% B. Accurate mass spectrometry was carried out using an Agilent Accurate Mass 6230 TOF apparatus. Dynamic mass axis calibration was achieved by continuous infusion, post-chromatography, of a reference mass solution using an isocratic pump connected to a multimode ionization source, operated in the positive-ion mode. ESI capillary and fragmentor voltages were set at 3,500 V and 100 V, respectively. The nebulizer pressure was set at 40 psi and the nitrogen drying gas flow rate was set at 10 L/min. The drying gas temperature was maintained at 250°C. The MS acquisition rate was 1.5 spectra/sec and *m*/*z* data ranging from 80-1,200 were stored. This instrument routinely enabled accurate mass spectral measurements with an error of less than 5 parts-per-million (ppm), mass resolution ranging from 10,000–25,000 over the *m*/*z* range of 121–955 atomic mass units, and a 100,000-fold dynamic range with picomolar sensitivity. Data were collected in the centroid mode in the 4 GHz (extended dynamic range) mode. Detected *m*/*z* were deemed to be identified metabolites on the basis of unique accurate mass-retention time identifiers for masses exhibiting the expected distribution of accompanying isotopomers (21035735). Typical variation in abundance for most of the metabolites stayed between 5 and 10% under these experimental conditions.

### ^15^N-labeling analysis

Under the experimental conditions described above, M+1 arising from ^15^N incorporation can be readily distinguished from M+1 arising from natural abundance ^13^C, therefore allowing direct monitoring of ^15^N labeling. The extent of ^15^N labeling for each metabolite was determined by dividing the summed peak height ion intensities of all ^15^N labeled species by the ion intensity of both labeled and unlabeled species, expressed in percent.

### Macrophages & infection procedure

Bone marrow cells were flushed from the femurs and tibias of 6–8 weeks old female C57BL/6 mice, and cultured in Petri dishes (2.10^6^ cells/dish) in RPMI 1640 GlutaMax (GIBCO) supplemented with 10% fetal calf serum (FCS, Pan-Biotech) and 20 ng/mL macrophage colony-stimulating factor (M-CSF, Peprotech) at 37°C in the presence of 5% CO_2_. At day 6, cells were transferred to 24-well plastic plates (2.10^5^ cells/well). For macrophage activation, cells were incubated with 10 ng/mL interferon gamma (IFNγ, Peprotech) and 5 ng/mL LPS (Invivogen) overnight prior to infection. Infection was performed in triplicate at a multiplicity of infection of 0.1 bacterium per cell for 4 h at 37°C. Cells were then washed 2 times with DPBS before addition of fresh medium. At day 0, 2 and 5, cells were lysed in 0.01% Triton X-100 (Sigma), and serial dilutions of the lysates were plated onto 7H11-OADC agar medium for CFU scoring. For infection experiments using Bafilomycin A1 (Sigma), cells were pre-incubated 1 h with 100 nM bafilomycin A1 prior to infection and removed at 24 h post-infection.

### Secondary ion mass spectrometry

SIMS analysis was carried out using a modified version of a previously described protocol [30]. Briefly, for ^13^C labeling, bacteria were grown in minimal medium containing 0.5 g/L KH_2_PO_4_, 0.5 g/L MgSO_4_, 15 mM NH_4_SO_4_, 10 g/L ^13^C glycerol supplemented with 0.05% tyloxapol (Sigma) and neutralized to pH 6.5–7.0 with NaOH before filtration. In order to overcome the difficulties encountered during the sample preparation stage in our previous experiments due to incomplete resin infiltration [30], in the present study cells were deposited directly on clean Silicon chips, and were infected and labeled. Macrophages were infected at a multiplicity of infection of 10 bacteria per cell with ^13^C labeled-bacteria for 4 hours. At 20 h post-infection, the culture medium was replaced by fresh RPMI containing 10% FCS and 5 mM ^15^N_1 (amine)_-asparagine. After 4 h at 37°C, cells were fixed with 4% PFA, 2.5% glutaraldehyde in a 0.1 M cacodylate buffer (pH 7.4). The detailed analytical conditions for SIMS imaging were described previously [30]. Briefly, a NanoSIMS-50 Ion microprobe (CAMECA, Gennevilliers, France) operating in scanning mode was used [66]. A Cs^+^ primary ion beam steps over the surface of the sample and four secondary ion species (^12^C^−^, ^13^C^−^, ^12^C^14^N^−^, ^12^C^15^N^−^) were monitored simultaneously to create images of these selected ion species. The identification of bacteria location was highlighted by high ^13^C content while the asparagine uptake was revealed by ^15^N enrichment. Prior to image acquisition, the upper layer of the cells was eroded away using high density primary Cs^+^ ion bombardment until the underlying structures with ^13^C-labeled bacteria could be observed. Consequently, analysis of ^15^N enrichment could be performed. The image acquisition was then carried out using multiframe mode. The primary beam intensity was 1 pA with a typical probe size of 100 nm (distance between 16%–84% of peak intensity from a line scan) and the raster size ranges from 40 to 50 µm in order to image a whole cell with an image definition of 512×512 pixels. With a dwell time of 2 ms per pixel, up to 25 frames were acquired and the total analysis time was 3 hours. Image treatment was performed using ImageJ software [67]. First, multiframe images were properly aligned using CN^−^ images as reference before a summed image was obtained for each ion species. A map of ^13^C atomic fraction was deduced from ^12^C^−^ and ^13^C^−^ images. In parallel, regions of interest were manually defined based on the ^13^C^−^ map so as to outline individual bacterium for data extraction. For ^15^N/^14^N ratio quantification, a sample containing no labeled cells was used as working reference for adjusting the detectors. Finally, the ^13^C map, as well as the one for ^15^N/^14^N ratio, are displayed in Hue-Saturation-Intensity (HSI) mode. These HSI color images were generated using OpenMIMS, an ImageJ plugin developed by Claude Lechene's Laboratory (http://nrims.harvard.edu/software) [68]. Although the cellular structures were less visible using this method compared to the ones obtained with thin sections of resin-embedded cells, we have shown in the previous study [30] that the results were similar, either for ^13^C labeling, or for ^15^N enrichment.

### Immuno-electron microscopy

Mouse bone marrow-derived macrophages infected with either the wild type strain of *M. tuberculosis* H37Rv or the recombinant strain over-expressing His-tagged AnsA were first fixed for 1 h at room temperature with a mixture of EM grade 2.5% paraformaldehyde (Euromedex) and 0.1% glutaraldehyde (Sigma) prepared in 0.1 M Na-cacodylate buffer, pH 7.2, containing 5 mM CaCl_2_, 5 mM MgCl_2_ and 0.1 M sucrose. Cells were then washed twice with the same buffer for 15 min each, once with the same buffer containing 50 mM NH_4_Cl for 15 min and once with the same buffer devoid of sucrose for 5 min. Cells were scraped off the culture dishes with a rubber policeman and concentrated in 1% agar prepared in the same buffer devoid of sucrose. Cells were then processed for embedding in Lowicryl HM20 using the PLT (progressive lowering of temperature) procedure [69]. High-resolution labeling of His-tagged AnsA was performed on thin sections (90 nm-thick) deposited onto carboned-coat nickel EM grids as previously described [70], [71]. Briefly, grids were sequentially floated on i) water for 5–10 min, ii) PBS-1% BSA for 5 min to block unspecific sites, iii) 5 nm Ni-NTA-Nanogold diluted 5-fold in PBS containing 0.5% BSA and 0.05% Tween 20 for 30 min, iv) 5 mM imidazole for 1 min, v) PBS for 3×1 min, and vi) distilled H_2_O for 2×5 min. All incubations were carried out at room temperature. Sections were either not stained or slightly stained for 30 sec with 1% uranyl acetate in distilled water, and observed under the electron microscope (Zeiss 912).

### Confocal microscopy & flow cytometry

Murine macrophages were prepared as described above, plated after 6 days of differentiation on cover glasses at 2.10^5^ cells/well in a 24-well plates, and activated with IFNγ (10 ng/mL) and LPS (5 ng/mL) overnight prior to infection. 10 mL of bacteria grown until OD_600_ of 1 in complete 7H9 were centrifuged for 7 min at 4,000 rpm, and washed two times with 20 mL DPBS. For bacteria labeling, pellets were suspended in 250 µL of Alexa Fluor 488 succinimidyl ester (Fisher) (1.5 µL Alexa Fluor 488 in 248.5 µL DPBS) or, for intra-phagosomal pH measurement, in 250 µL Alexa Fluor 647-NHS ester and 5-carboxyfluorescein succinimidyl ester (Fisher) (1.5 µL each in 247 µL DPBS), and incubated at room temperature for 45 min. Bacteria were then washed two times with 20 mL DPBS and disaggregated manually for 30 sec with sterile glass beads. Bacteria were then resuspended in 7 mL of complete RPMI and centrifuged for 5 min at 1,200 rpm to remove aggregates. The OD_600_ of bacterial suspensions was then measured to determine the number of bacteria. Cells were infected at an MOI of 10 bacteria/cell for 1 h at 37°C and washed two times with DPBS before addition of fresh medium. After 1 and 3 h infection, cells were stained with 1 µM LysoTracker Red DND-99 (Molecular Probes) in complete RPMI for 1 h, washed with DPBS and fixed for 2 h with PFA 4% at room temperature. For immuno-detection of the V-ATPase, a rabbit polyclonal antibody (Synaptic Systems) was used at 1/100 dilution. Cover glasses were then mounted on glass slides using a VECTASHIELD Hardset Mounting Medium with DAPI (Cliniscience) and stored overnight at 4°C. Images were acquired with an LSM710 microscope equipped with a 40× 1.30 NA objective (Carl Zeiss, Inc.), recorded with Zen software (Carl Zeiss, Inc.), and analyzed with ImageJ software. All images were acquired with the same confocal microscope settings. The LysoTracker or V-ATPase signal intensity of every phagosome was measured with ImageJ software and the same threshold was applied for each condition to count the proportions of LysoTracker- or V-ATPase-positive phagosomes. See Figure 3 and S6 for examples of phagosomes that were considered positive for LysoTracker and V-ATPase, respectively. Quantification of LysoTracker- and V-ATPase-positive phagosomes was realized for ≈300 phagosomes per condition. For phagosomal pH measurement, we used a modified version of a protocol we previously described [72]. Briefly, macrophages were pulsed with the dual dye-coupled (Alexa Fluor 647, 5-Carboxyfluorescein) mycobacteria (MOI 50) for 15 min and washed 3 times with PBS. The cells were then incubated at 37°C for the indicated times and immediately analyzed by FACS, using a gating FSC/SSC selective for macrophages. The ratio of the mean fluorescence intensity (MFI) emission between the two dyes was determined. Values were compared with a standard curve obtained by resuspending the cells that had phagocytosed labeled bacteria for 1 h at a fixed pH (ranging from pH 5.7 to 7.3) and containing 0.1% Triton X-100. Cells were immediately analyzed by FACS to determine the emission ratio of the two fluorescent probes at each pH value.

### Mouse infection

All animal experiments were performed in animal facilities that meet all legal requirements in France and by qualified personnel in such a way to minimize discomfort for the animals. All procedures including animal studies were conducted in strict accordance with French laws and regulations in compliance with the European community council directive 68/609/EEC guidelines and its implementation in France. All protocols were reviewed and approved by the Comité d'Ethique Midi-Pyrénées (reference MP/04/26/07/03). Six- to eight-week-old female C57BL/6 mice were anesthetized with a cocktail of ketamine (60 mg/kg; Merial) and xylasine (10 mg/kg; Bayer) and infected intranasally with 1,000 CFUs of the various mycobacterial strains in 25 µL of PBS-0.01% Tween 80. At 21 days post-infection, five mice per strain tested were sacrificed and lung and spleen homogenates were plated onto 7H11 agar plates for CFU scoring.

## Supporting Information

Figure S1 **A genetic strategy to construct the** ***ansA*** **-KO mutant in** ***M. tuberculosis*** **, including the assessment of its essentiality.** (**a**) Genetic organization of the *ansA* locus in *M. tuberculosis*. (**b**) We first generated a merodiploid strain harboring an additional copy of *ansA* at the phage recombination site *attL5* [43]. (**c**) The original *ansA* gene was replaced by a kanamysin-resistance cassette through recombination. (**d**) Exogenously inserted *ansA* allele was replaced by a zeocin resistance cassette.
(JPG)

Figure S2 **Growth of** ***ansA*** **-KO with asparagine or glutamate as sole nitrogen source.** Growth of *M. tuberculosis* H37Rv or the *ansA*-KO mutant strains in minimal medium containing 5 mM asparagine (ASN) or 5 mM glutamate (GLU) as sole nitrogen source. Growth was measured by monitoring turbidity; data represent mean±s.d. of triplicate samples and are representative of two independent experiments.
(JPG)

Figure S3 **Asparagine supports** ***M. tuberculosis*** **growth mostly through providing nitrogen.** Growth of *M. tuberculosis* H37Rv in minimal medium containing 50 mM asparagine (Asn), 50 mM asparagine and 15 mM ammonium (Asn + NH_4_^+^) or 50 mM asparagine and 10 g/L glycerol (Asn + Gro). Growth was measured by monitoring turbidity; data represent mean±s.d. of triplicate samples and are representative of at least three independent experiments.
(JPG)

Figure S4 **AnsA is essential for nitrogen assimilation from asparagine at acidic pH.** (A) Frequency of ^15^N-glutamate (GLU) and ^15^N-glutamine (GLN) detected in *M. tuberculosis* wild type (H37Rv), the *ansA*-KO mutant and its complemented strain (Compl.) cultivated in minimal medium in the presence of 2 mM ^15^N-asparagine as sole nitrogen source at pH 5.5. Data represent mean±s.d. of triplicate samples and are representative of at least two independent experiments. #, not detected. (B–D) Same experiment as in Fig. 3E,F with a dense bacterial suspension (OD_600_ = 1.5), and with asparagine (B,C) or aspartate (D) as sole nitrogen source.
(JPG)

Figure S5 **AnsP2 is not involved in** ***M. tuberculosis*** **intracellular survival.** IFNγ- and LPS-activated mouse bone marrow-derived macrophages were infected with *M. tuberculosis* wild type (H37Rv) or the *ansP2*-KO mutant at a multiplicity of infection of 0.1 bacterium/cell for 4 h at 37°C. Cells were washed and further incubated with fresh medium for 0, 2 or 5 days. At the indicated time-points, cells lysates were plated for CFU scoring.
(JPG)

Figure S6 **V-ATPase accumulates in phagosomes containing the** ***M. tuberculosis ansA*** **-KO mutant.** IFNγ- and LPS-activated mouse bone marrow-derived macrophages were infected with *M. tuberculosis* wild type (H37Rv), the *ansA*-KO mutant or the complemented strain at a multiplicity of infection of 0.1 bacterium/cell for 4 h at 37°C. Cells were washed, fixed, stained with an anti-V-ATPase antibody and a Texas Red-coupled secondary antibody, and processed for confocal microscopy analysis (A). Bar represents 10 µm. Arrowheads point to example phagosomes considered positive for V-ATPase staining. (B) Colocalisation events were recorded in ≈300 phagosomes observed in ≈10 different fields. Data are representative of three independent experiments. In (B), data represent mean±s.d. of phagosomes recorded in one representative experiment, and were analyzed using the Student's *t* test; **, P<0.01.
(JPG)
