# Supplementary figures and images for: Mycobacterium tuberculosis Exploits Asparagine to Assimilate Nitrogen and Resist Acid Stress during Infection
Source: PLoS Pathog. 2014 Feb 20;10(2):e1003928. doi: 10.1371/journal.ppat.1003928 (PMC3930563; doi:10.1371/journal.ppat.1003928)

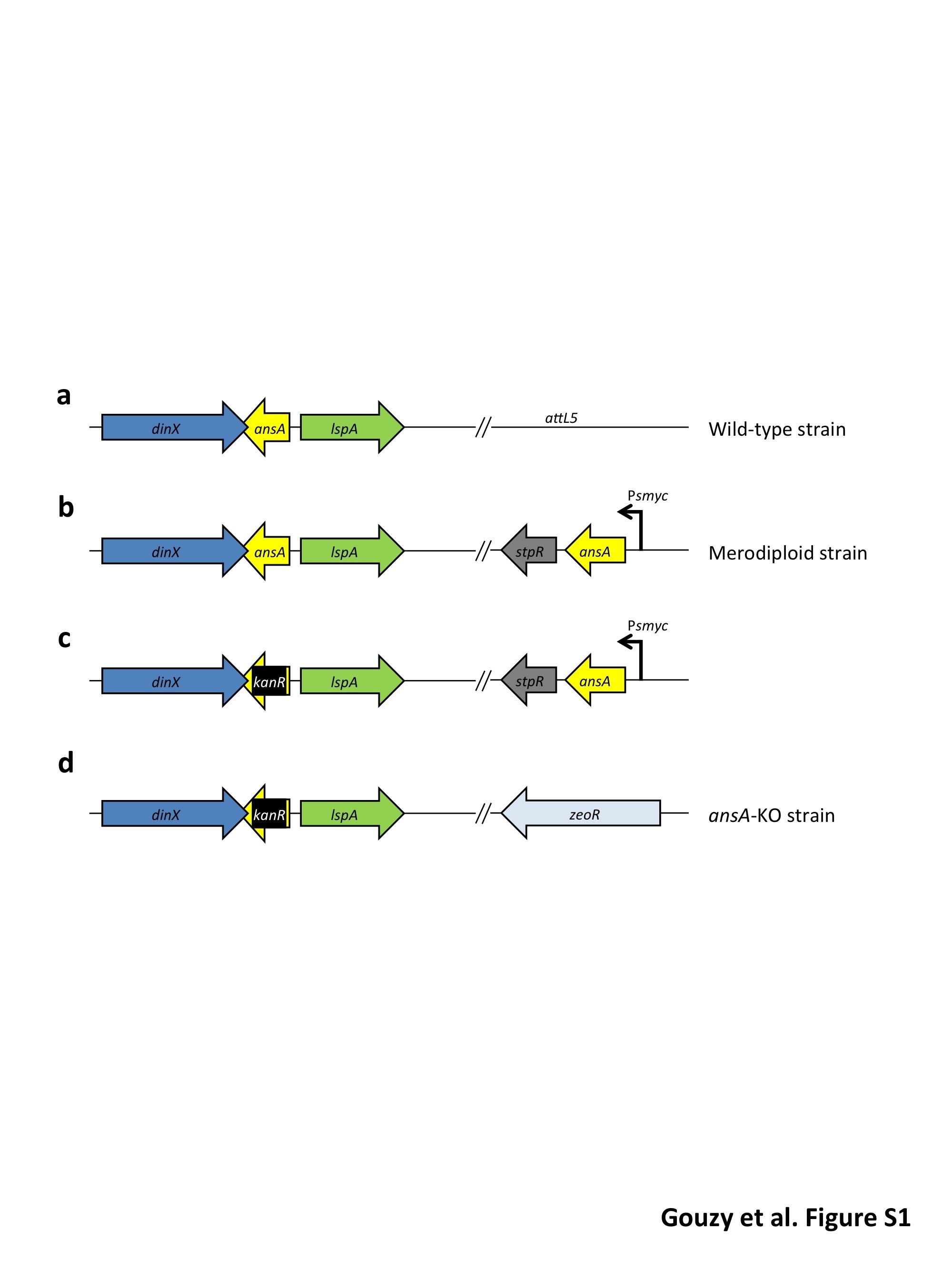

Supplement: Figure S1 — A genetic strategy to construct the ansA -KO mutant in M. tuberculosis , including the assessment of its essentiality. (a) Genetic organization of the ansA locus in M. tuberculosis. (b) We first generated a merodiploid strain harboring an additional copy of ansA at the phage recombination site attL5 [43]. (c) The original ansA gene was replaced by a kanamysin-resistance cassette through recombination. (d) Exogenously inserted ansA allele was replaced by a zeocin resistance cassette. (JPG) [file ppat.1003928.s001.jpg]

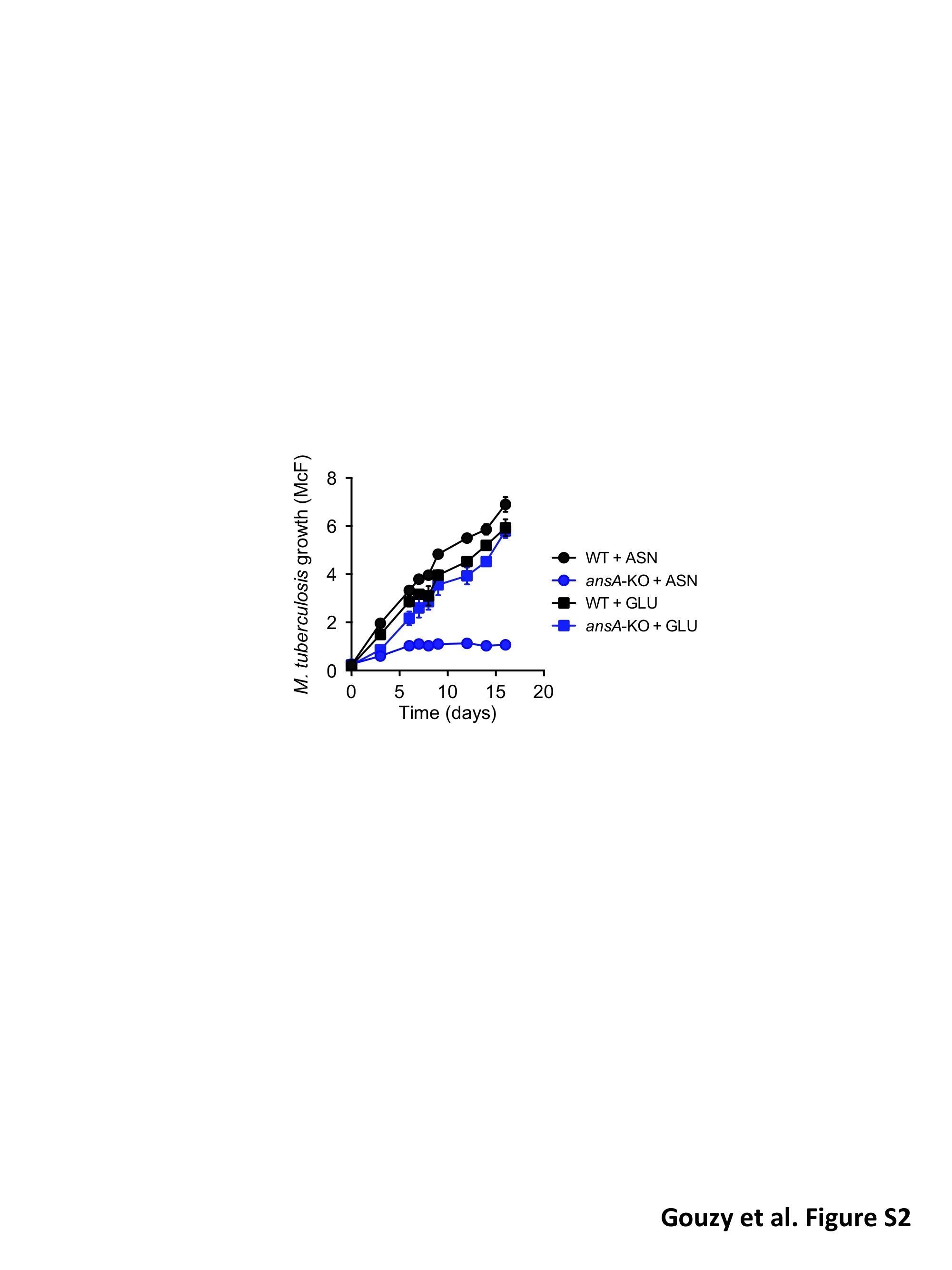

Supplement: Figure S2 — Growth of ansA -KO with asparagine or glutamate as sole nitrogen source. Growth of M. tuberculosis H37Rv or the ansA-KO mutant strains in minimal medium containing 5 mM asparagine (ASN) or 5 mM glutamate (GLU) as sole nitrogen source. Growth was measured by monitoring turbidity; data represent mean±s.d. of triplicate samples and are representative of two independent experiments. (JPG) [file ppat.1003928.s002.jpg]

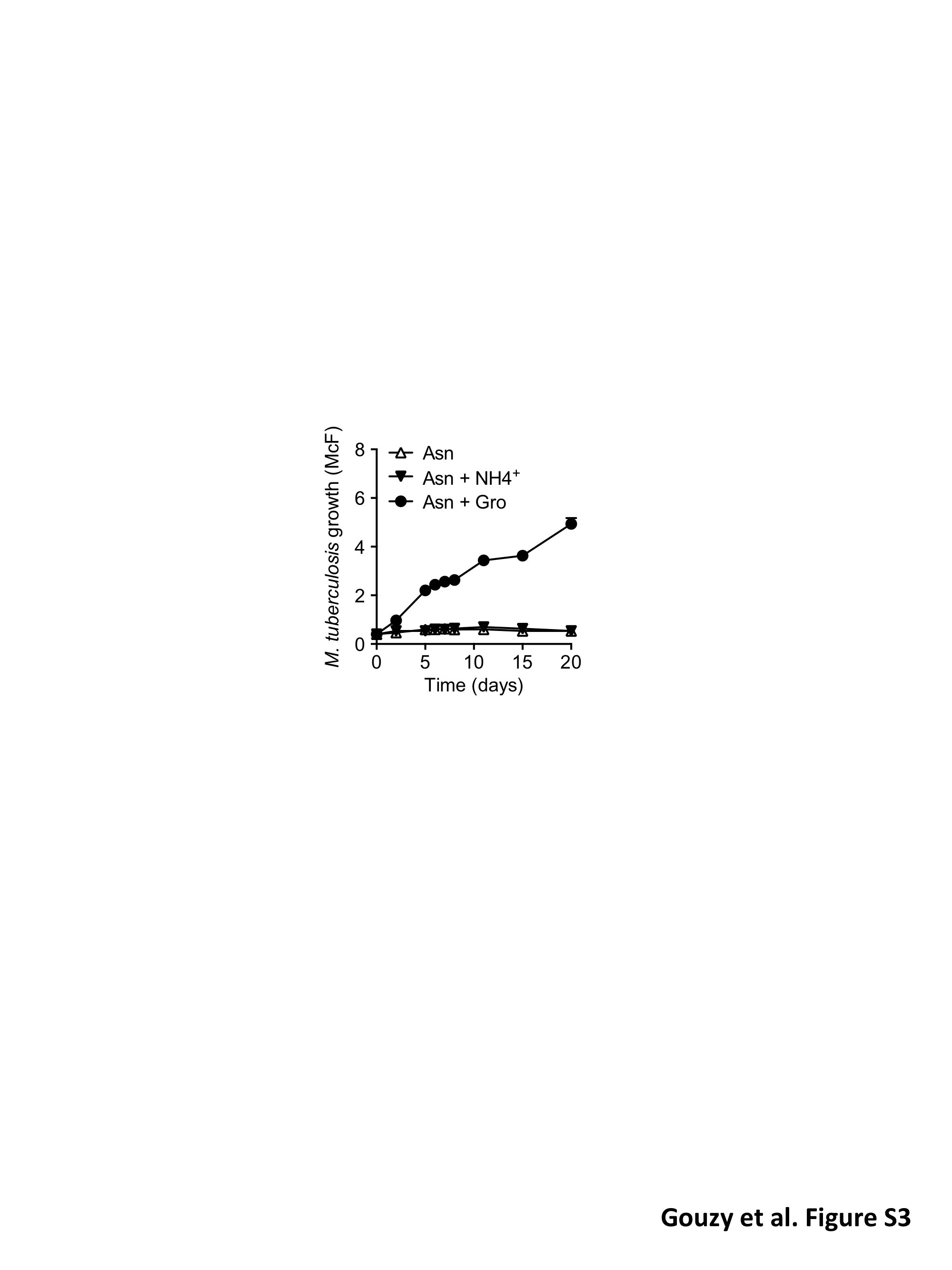

Supplement: Figure S3 — Asparagine supports M. tuberculosis growth mostly through providing nitrogen. Growth of M. tuberculosis H37Rv in minimal medium containing 50 mM asparagine (Asn), 50 mM asparagine and 15 mM ammonium (Asn + NH4 +) or 50 mM asparagine and 10 g/L glycerol (Asn + Gro). Growth was measured by monitoring turbidity; data represent mean±s.d. of triplicate samples and are representative of at least three independent experiments. (JPG) [file ppat.1003928.s003.jpg]

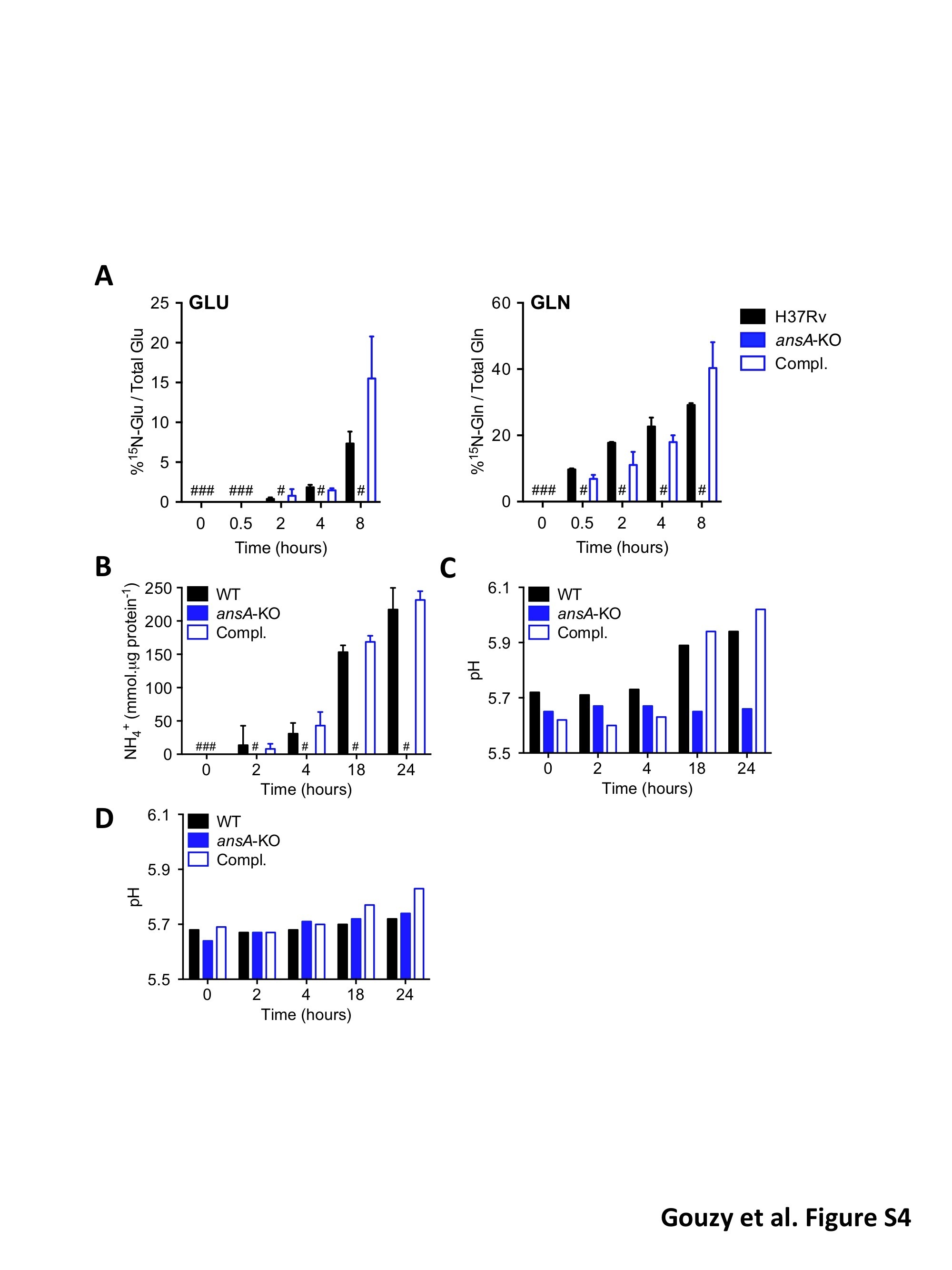

Supplement: Figure S4 — AnsA is essential for nitrogen assimilation from asparagine at acidic pH. (A) Frequency of 15N-glutamate (GLU) and 15N-glutamine (GLN) detected in M. tuberculosis wild type (H37Rv), the ansA-KO mutant and its complemented strain (Compl.) cultivated in minimal medium in the presence of 2 mM 15N-asparagine as sole nitrogen source at pH 5.5. Data represent mean±s.d. of triplicate samples and are representative of at least two independent experiments. #, not detected. (B–D) Same experiment as in Fig. 3E,F with a dense bacterial suspension (OD600 = 1.5), and with asparagine (B,C) or aspartate (D) as sole nitrogen source. (JPG) [file ppat.1003928.s004.jpg]

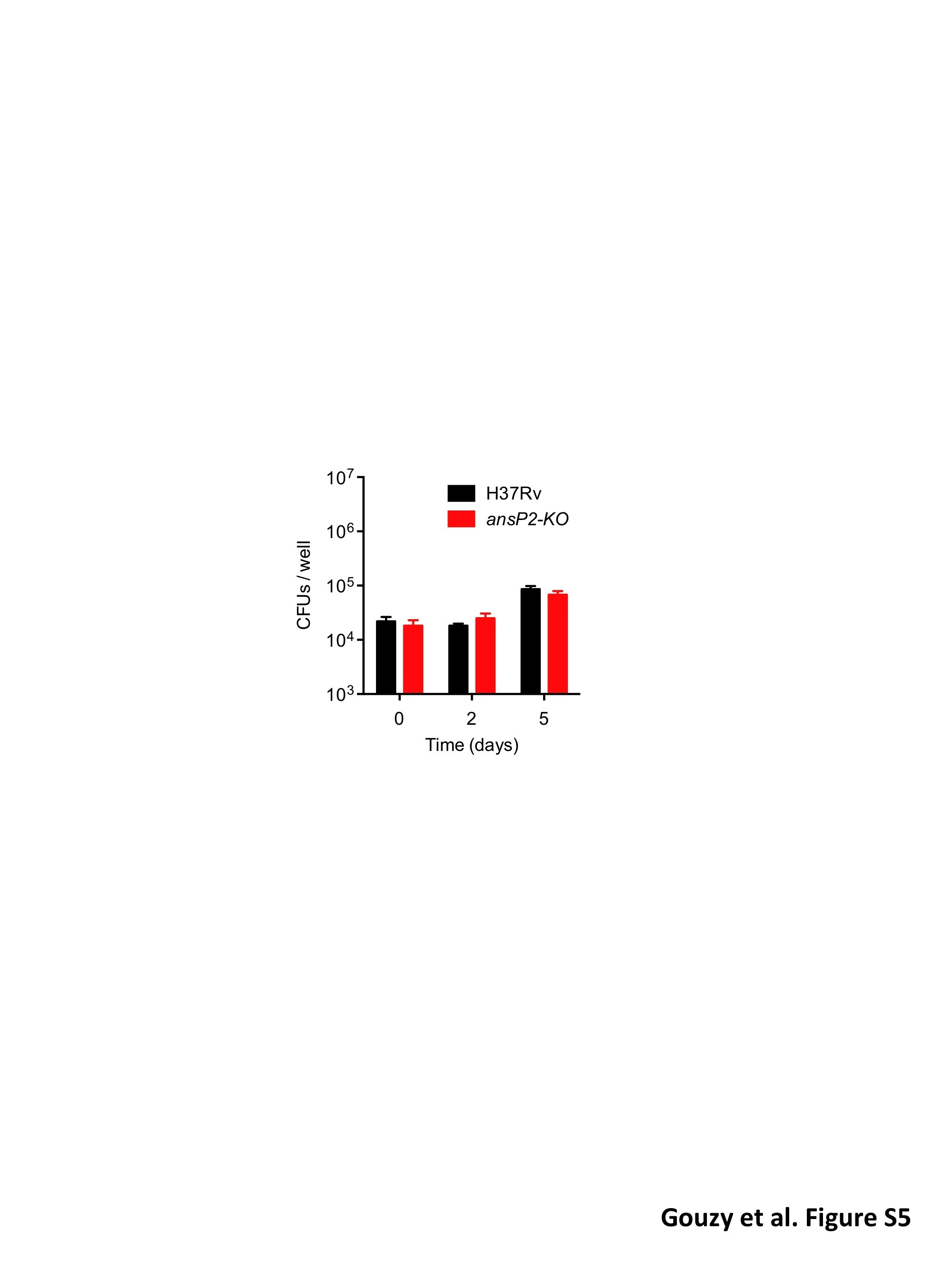

Supplement: Figure S5 — AnsP2 is not involved in M. tuberculosis intracellular survival. IFNγ- and LPS-activated mouse bone marrow-derived macrophages were infected with M. tuberculosis wild type (H37Rv) or the ansP2-KO mutant at a multiplicity of infection of 0.1 bacterium/cell for 4 h at 37°C. Cells were washed and further incubated with fresh medium for 0, 2 or 5 days. At the indicated time-points, cells lysates were plated for CFU scoring. (JPG) [file ppat.1003928.s005.jpg]

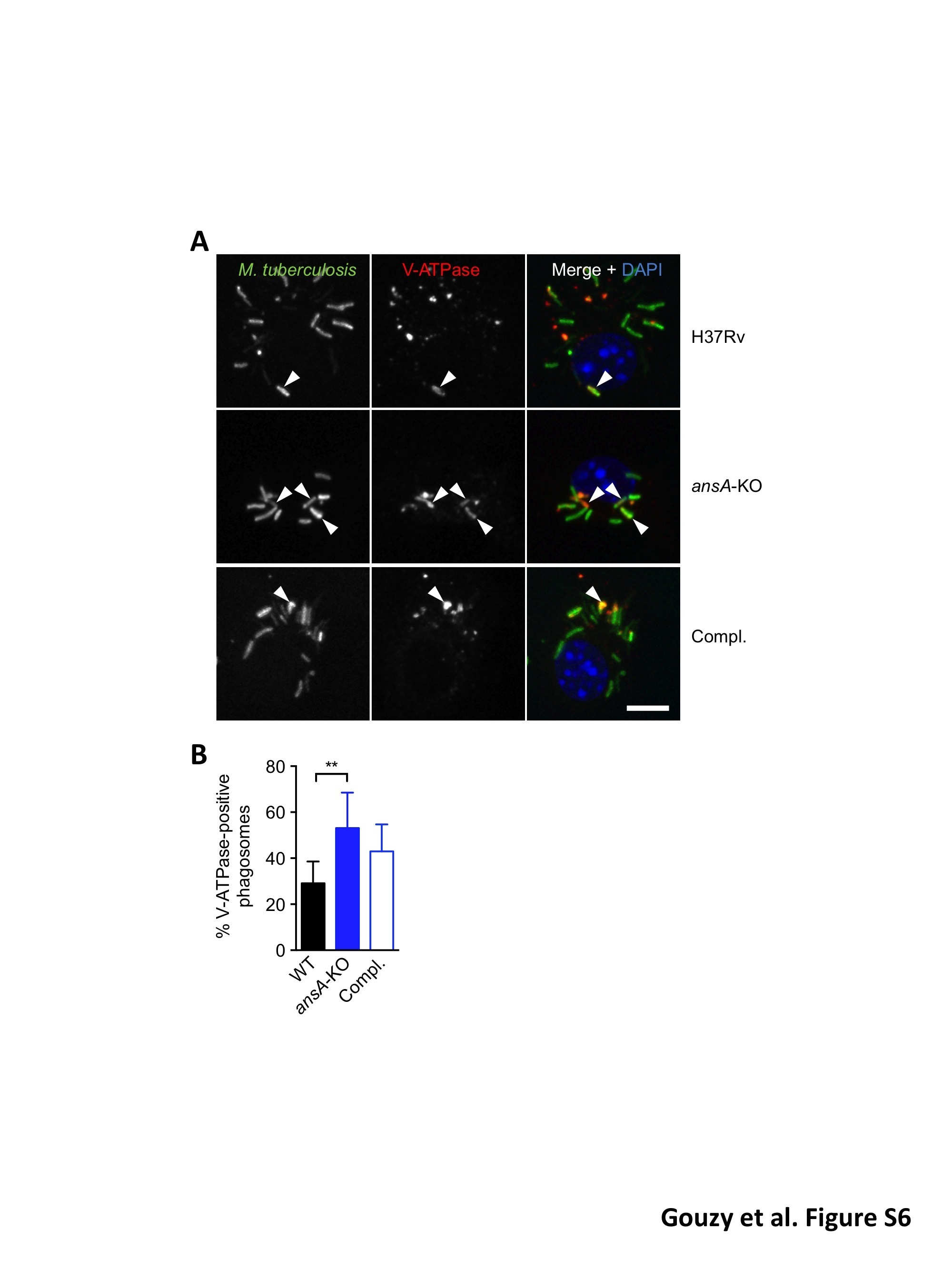

Supplement: Figure S6 — V-ATPase accumulates in phagosomes containing the M. tuberculosis ansA -KO mutant. IFNγ- and LPS-activated mouse bone marrow-derived macrophages were infected with M. tuberculosis wild type (H37Rv), the ansA-KO mutant or the complemented strain at a multiplicity of infection of 0.1 bacterium/cell for 4 h at 37°C. Cells were washed, fixed, stained with an anti-V-ATPase antibody and a Texas Red-coupled secondary antibody, and processed for confocal microscopy analysis (A). Bar represents 10 µm. Arrowheads point to example phagosomes considered positive for V-ATPase staining. (B) Colocalisation events were recorded in ≈300 phagosomes observed in ≈10 different fields. Data are representative of three independent experiments. In (B), data represent mean±s.d. of phagosomes recorded in one representative experiment, and were analyzed using the Student's t test; **, P<0.01. (JPG) [file ppat.1003928.s006.jpg]
